# Advancing forensic-based investigation incorporating slime mould search for gene selection of high-dimensional genetic data

**DOI:** 10.1038/s41598-024-59064-w

**Published:** 2024-04-13

**Authors:** Feng Qiu, Ali Asghar Heidari, Yi Chen, Huiling Chen, Guoxi Liang

**Affiliations:** 1 Institute of Big Data and Information Technology, Wenzhou University, Wenzhou, 325035 China; 2 School of Surveying and Geospatial Engineering, College of Engineering, University of Tehran, Tehran, Iran; 3 Department of Computer Science and Artificial Intelligence, Wenzhou University, Wenzhou, 325035 China; 4 Department of Artificial Intelligence, Wenzhou Polytechnic, Wenzhou, 325035 China

**Keywords:** Forensic-based investigation, High-dimensional genetic data, Gene selection, Slime mould algorithm, Global optimization, Computational science, Computer science

## Abstract

Modern medicine has produced large genetic datasets of high dimensions through advanced gene sequencing technology, and processing these data is of great significance for clinical decision-making. Gene selection (GS) is an important data preprocessing technique that aims to select a subset of feature information to improve performance and reduce data dimensionality. This study proposes an improved wrapper GS method based on forensic-based investigation (FBI). The method introduces the search mechanism of the slime mould algorithm in the FBI to improve the original FBI; the newly proposed algorithm is named SMA_FBI; then GS is performed by converting the continuous optimizer to a binary version of the optimizer through a transfer function. In order to verify the superiority of SMA_FBI, experiments are first executed on the 30-function test set of CEC2017 and compared with 10 original algorithms and 10 state-of-the-art algorithms. The experimental results show that SMA_FBI is better than other algorithms in terms of finding the optimal solution, convergence speed, and robustness. In addition, BSMA_FBI (binary version of SMA_FBI) is compared with 8 binary algorithms on 18 high-dimensional genetic data from the UCI repository. The results indicate that BSMA_FBI is able to obtain high classification accuracy with fewer features selected in GS applications. Therefore, SMA_FBI is considered an optimization tool with great potential for dealing with global optimization problems, and its binary version, BSMA_FBI, can be used for GS tasks.

## Introduction

With the rapid development of modern information technology and biomedical fields, researchers can study biomedical fields at the genetic level^1–3^. DNA microarray technology is a technique that enables the rapid and efficient detection and analysis of the expression levels of millions of genes^4,5^. These gene expression data are an essential source of information for studying the current physiological state of cells, and these data have been widely used for early diagnosis and prognosis prediction of diseases. The microarray technology shines as an emerging and valuable technology in generating high-dimensional data from a few samples^6,7^. However, for this type of dataset, the high feature dimensionality makes it prone to dimensional catastrophe; at the same time, the relatively small sample size causes the algorithm to be prone to overfitting, making it difficult to extract accurate patterns and correlations from it. Therefore, feature selection (FS) is performed for gene data to reduce the data dimensionality, i.e., gene selection (GS) is a method to solve this problem.

Advances in healthcare information systems require more efficient algorithms to process the various data in the system, and techniques such as machine learning and FS are well used in this field^8–10^. FS of gene data is becoming more prominent as a screening method to reduce irrelevant microarray gene data and improve classification accuracy. FS efficiently identifies and extracts the most valuable features from a large data set^11,12^. This approach allows researchers and analysts to focus on the most relevant and informative features, which improves the accuracy and efficiency of subsequent analyses and models and helps to make informed decisions in different disciplines, such as genetics and biology. In reducing the number of features, elimination of redundant and invalid features plays a vital role and is especially beneficial in dealing with high-dimensional datasets^13,14^. As the number of features in a dataset increases, model training becomes more challenging. Unnecessary features can significantly lengthen the training time and negatively affect the model's performance. Therefore, FS prior to training can improve the effectiveness of the model. Especially in high-dimensional genetic datasets, FS is an essential step because it provides a valuable way to reduce unnecessary features.

FS is a crucial technique in data processing and machine learning. Its main goal is to select the best feature subset from the original data, thus reducing the dimensionality of the data. Based on different approaches, FS can be categorized into five types: filtering, wrapping, embedding, ensemble, and hybrid^15,16^. Among them, wrapping methods rely on classification algorithms to select a subset of features and can achieve more desirable results.

Current optimization algorithms mainly include the exact solution method and approximate solution method^17^. The exact solution method is usually applied to optimization problems with small problem sizes and relatively simple structures. However, due to the limitation of computational complexity, it may face computational difficulties when dealing with large-scale and complex problems. Approximate solution algorithms can find an approximate optimal solution in a fair time, and this class of methods is more suitable for large-scale and complex problems. Metaheuristic algorithms (MAs) are an important branch of approximate solution methods, and they have also been one of the most active research areas in computer science in the past few years^18^. In recent years, researchers have proposed a large number of MA, including Differential Evolution (DE)^19^, Bat Algorithm (BA)^20^, Grasshopper Optimization Algorithm (GOA)^21^, Colony Predation Algorithm (CPA)^22^, weighted meaN oF vectOrs (INFO)^23^, Harris Hawk Algorithm (HHO)^24^, Sine Cosine Algorithm (SCA)^25^, Ant Colony Algorithm (ACO)^26^, Runge Kutta Optimizer (RUN)^27^, Slime Mould Algorithm (SMA)^28^, Gravity Search Algorithm (GSA)^29^, Hunger Games Search (HGS)^30^, Rime Optimization algorithm (RIME)^31^, Parrot Optimizer (PO)^32^, and Liver Cancer Algorithm (LCA)^33^, among others. These methodologies are aimed at solving problems across various fields, such as networking^34,35^, image segmentation^36^, and the Internet of Things^37,38^, among others. As optimization problems become more complex, there is a need for more intelligent algorithms that can use their exploration and exploitation capabilities to deal with problems that are nonlinear and contain multiple locally optimal solutions.

Recently, researchers have proposed several hybrid techniques incorporating different search strategies for feature selection problems, providing suitable references and ideas for this paper. Hussain et al.^39^ proposed a hybrid optimization approach integrating the sine–cosine algorithm into HHO while dynamically adjusting the candidate solutions to avoid the solution stagnation problem in HHO. Through testing on the CEC 2017 test set as well as 16 feature selection datasets containing both low and high dimensions, the results demonstrate that the proposed hybrid variant of HHO produces efficient search results without increasing the computational cost. A new variant of SSA for the feature selection problem is introduced by Neggaz et al.^40^. The authors use a sinusoidal mathematical function inspired by the sine–cosine algorithm to update the positions of the followers in the SSA while using a disrupt operator to increase the diversity of the population. Evaluation of 20 datasets containing 4 high dimensions demonstrates that the algorithm performs well in accuracy, sensitivity, specificity, and number of selected features. Building on previous work, our study focuses on high-dimensional gene selection data, so the selected datasets are almost all high-dimensional small-sample gene data.

The investigation-localization-tracking process is often involved in criminal investigations by police officers involved in criminal investigations. Inspired by this process, Chou et al.^41^ proposed a Forensic-based Investigation (FBI) algorithm in 2020. FBI has several advantages, such as no predefined parameters, good performance, robustness, and stability. As a result, it has received much attention. It has been successfully used in diverse fields, including resource-constrained scheduling problems^41^, solar cell models^42^, framework hyperparameter optimization^43^, optimal parameters for fuel cell MPPT^44^, and lateral vibration of ground-based systems^45^, and edge smart grids^46^. In this study, an FBI-based algorithm will be used for FS.

However, like other MAs, the original FBI has shortcomings in solving high-dimensional data and real-world problems. Therefore, to alleviate the problems and balance the exploitation and exploration capabilities of the FBI, many improved variants of the FBI have been proposed recently. Kaveh et al.^47^ proposed a frequency-constrained dome truss optimization design method based on Enhanced Forensic Investigations (EFBI), which modifies the original FBI to enhance the link between the investigation and tracing teams in the algorithm. EFBI is used in three dome trusses with frequency constraints optimization problems, and comparisons are made with other optimization algorithms to verify the improvement of the improved algorithm. Kuyu et al.^48^ focused on the search process of the FBI algorithm, i.e., Step A and Step B stages. The algorithm's behavior is improved by enhancing the diversity of the population by incorporating dyadic-based learning in Stage A and integrating the Cauchy variation mechanism in Step B to improve the ability to jump out of the local optimal solution. Validated on two benchmark test sets and six real-world problems, the dyadic-based learning and the Cauchy variation mechanism positively affect the FBI. Malika et al.^49^ suggested a quasi-oppositional forensic investigation (QOFBI)-inspired approach for producing optimal outcomes for DG allocation and sizing that incorporates all the system's operating limitations. To demonstrate the enhanced capabilities of the suggested method, IEEE 33-bus, IEEE 69-bus, and IEEE 85-bus test systems are simulated. Several metrics of performance for power, voltage, and stability have been calculated for various degrees and kinds of DG penetration, and a comparative study finding with the previously recommended strategy are provided and examined. The outcomes illustrate that the approach is more robust and efficient, and it operates superior with less computing cost.

Nguyen et al.^50^ devised a framework integrating Building Information Modeling (BIM), Multi-Objective Optimization (MOO), and Multi-Criteria Decision Making (MCDM) to address resource tradeoffs in project scheduling. Initially, BIM construction management software is leveraged to develop a comprehensive 3D model, facilitating the generation of a quantity list delineating the necessary project resources. A novel approach, termed Multi-Objective Forensic Investigation Method (MOFBI), is introduced to achieve the most favorable outcome. Subsequently, an evidence-driven multi-criteria decision analysis technique is applied to ascertain the ideal project execution schedule. The efficacy and potency of this framework are authenticated through the examination of three distinct project scheduling challenges. Tolba et al.^51^ presented a robust, improved forensic-based investigation (mFBI) optimization method for calculating the most efficient location of distributed generators (DGs) in electricity distribution networks (EDNs) to minimize the loss of power, as well as voltage deviations. Furthermore, hierarchical analysis is employed to derive the most relevant weighting factors for the multi-objective function (MOF). The efficacy of the proposed mFBI technique is validated and demonstrated through an investigation into the impact of DG integration on 118 IEEE EDN nodes and real Delta-Egypt EDN nodes. Chou et al.^52^ suggested a forensic-based multi-objective investigation method for the multi-objective engineering optimization problem. Within this algorithm, the population undergoes initialization via chaotic mapping. Subsequently, Lévy flights, two elite groups, and a fixed-size file are employed to regulate the activities of investigators and police officers during offender search and frisking procedures. Simultaneously, a control time mechanism is integrated into MOFBI to harmonize exploration and exploitation, thereby attaining a Pareto-optimal solution within the multi-objective search space. Experiments show that MOFBI can approximate the Pareto-optimal frontier more accurately than other algorithms.

Although many advanced and improved FBIs have been proposed, most of the existing improved algorithms still suffer from the question of slow convergence and the greater probability of falling into local optimal when solving some specific cases. In the original FBI algorithm, two phases are included in the process of criminal investigation: one is the investigation phase, and the other is the tracing phase. The two phases perform independent searches in their respective populations, and trapping into the same local optimal is possible. The search agent generated by the SMA can adaptively go beyond the local optimal and better find the optimal solution through the positive and negative feedback mechanism. This article is based on the original FBI version, and the search phase of the slime mould algorithm has been added to assist with the solution. During the proposed algorithm, the slime mould search mechanism is integrated as an independent inspector group that compensates for the shortcomings of the investigation and pursuit groups. The slime mould search mechanism dynamically adjusts the search patterns according to the probability of the suspect being at the location. When the probability is high, the slime mould search mechanism uses an area-restricted search methodology, which focuses on the identified area. If the probability of the suspect being at the location is initially found to be low, the slime mould search mechanism controls the search to jump out of the current area and look for other locations with a high probability of the suspect being there. Thus, this strategy can significantly increase the convergence speed of the algorithm and the capability of skipping local optimal. A new variant of the FBI, called SMA_FBI, is developed by incorporating the SMA strategy into the original FBI. After that, the binary version of the algorithm, i.e., BSMA_FBI, is obtained utilizing a conversion function, which is applied to the GS problem with high-dimensional data.

The remainder of this paper is divided into two sections: “Overview (FBI and SMA)” section describes the original FBI and SMA. In “Proposed SMA_FBI” section, SMA_FBI and the algorithm's time complexity are given a detailed description. “Experiments” section gives and analyzes the experimental results. “Discussion” section discusses the experiments as well as the results. “Conclusion and future work” section summarizes the conclusions and gives some future directions.

## Overview (FBI and SMA)

This section provides a detailed description of the FBI and SMA.

### Forensic-based investigation (FBI)

**Figure 1:**
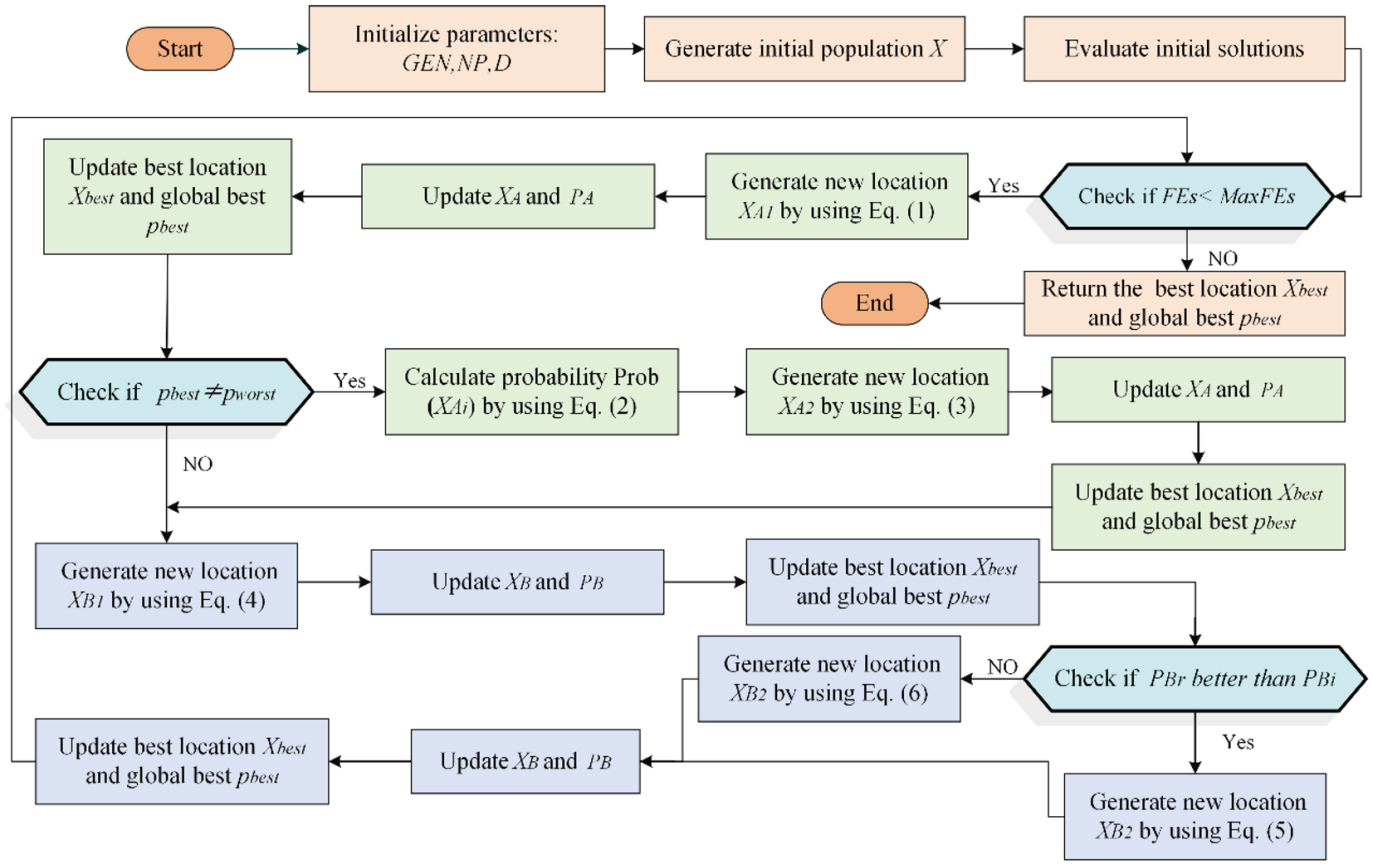
The flowchart of FBI.

The forensic-based investigation algorithm was inspired by Chou et al. from the investigation-localization-pursuit process of pursuing suspects by police officers involved in criminal investigations. It consists of two phases: the investigation phase (Step A) and the pursuit phase (Step B). The investigation phase is responsible for determining the location interval of the suspect in a general direction, while the pursuit phase requires a detailed search at the suspect location. The search space of the algorithm is defined as all possible suspect locations with the probability of locating the suspect as a metaphor for the objective function. In the search space, the investigator analyzes and evaluates the collected information to determine the identity and location of suspects, based on which the police can carry out an arrest. During this process, investigators and pursuers shift the direction of the search based on the latest evidence, thus requiring them to coordinate closely with each other throughout the process. The pseudo-code of the FBI appears in Algorithm1. The flowchart for the FBI is pictured in Fig. 1.

The original FBI can be categorized into several imperative steps:

*Step A1* Interpretation of Discovered Information. During this step, the investigative team analyzes the collected findings and initially identifies possible suspect locations, which can be inferred based on information related to $X_{{A_{i} }}$ and other suspect points. In this work, each individual is influenced by the others. The new suspected location $X_{{A1_{i} }}$ is represented in Eq. (1).

1 $$X_{{A1_{ij} }} = { }X_{{A_{ij} }} + \left( {r_{1} - 0.5} \right){*}2){*}\left( {X_{{A_{ij} }} - \left( {X_{{A_{kj} }} + X_{{A_{hj} }} } \right)/2} \right)$$

where $X_{{A_{i} }}$ denotes the $i$ th suspicious point,$i = 1,2, \ldots ,NP$; $NP$ is the overall size of the population. $j = 1,2, \ldots ,D$; $D$ is the number of dimensions. $r_{1}$ represents a random number in the range of [0,1], $(\left( {r_{1} - 0.5} \right) *2$ indicates a random value between -1 and 1. $k,h,j$ are the three randomly selected suspicious locations,$\left\{ {k,h,j} \right\} \in \left\{ {1,2, \ldots ,NP} \right\}$.

*Step A2* Determine the direction of the investigation. To establish the most likely suspect site, the investigator compares the probability of each suspect location to each other. $p_{{A_{i} }}$ indicates the likelihood (objective value) that the suspect is located at position $X_{{A_{i} }}$, i.e., $p_{{A_{i} }}$ denotes the objective value for the location $X_{{A_{i} }}$ (i.e., $p_{{A_{i} }}$ = fobjective($X_{{A_{i} }}$)). The investigator evaluates the likelihood of the new suspect's position and compares it to the location of the current entrance. The site with the higher probability (objective value) of the suspect's presence will be reserved, while the other location will be discarded. The probability of each location is calculated by Eq. (2).

2 $$Prob\left( {X_{{A_{i} }} } \right) = \left( {p_{worst} - { }p_{{A_{i} }} } \right)/\left( {p_{worst} - p_{best} } \right)$$

where $p_{worst}$ is the minimum probability of the existence of a suspect, $p_{best}$ is the maximum probability, and $X_{best}$ is the optimal location. The update of the search position will be affected by other suspicious positions, the direction of random selection is introduced on the basis of the optimal individual $X_{best}$, to increase the diversity of the search area and expand the search space. The position update formula is shown below:

3 $$X_{{A2_{ij} }} = { }X_{best} + X_{{A_{dj} }} + r_{2} {*}\left( {X_{{A_{ej} }} - X_{{A_{fj} }} } \right)$$

where $X_{best}$ indicates the best position obtained in step A1, $r_{2}$ is a random value between 0 and 1; and $d,e,f$ are three random values indicating the three suspicious positions.

*Step B1* Begin the operation. In this stage, the arresting officer approaches the target location and arrests the suspect based on the best location provided by the investigation team. Each $B_{i}$ (pursuing officer) approaches the location with the best likelihood and updates the location if the newly approached location yields a better likelihood than the likelihood of the old location.

4 $$X_{{B1_{ij} }} = r_{3} {* }X_{{B_{ij} }} + r_{4} {*}\left( {X_{best} - X_{{B_{ij} }} } \right)$$

where $r_{3}$ and $r_{4}$ express two random values in the range of 0 and 1.$j = 1,2, \ldots ,D.$

*Step B2* Real-time position updates based on actions. While the tracking team is taking pursuit actions, it reports new suspect points to the headquarters in real-time. The headquarters will update the position and direct the tracking team to approach the suspect point. Each tracking team member works closely and interacts with each other. Agent $B_{i}$ approaches the target point and receives influence from team member $B_{r}$ at the same time. When the likelihood of $B_{r}$ is greater than the likelihood of $B_{i}$, a new suspect location is generated according to Eq. (5); and vice versa according to Eq. (6).

5 $$X_{{B2_{ij} }} = X_{{B_{rj} }} + { }r_{5} {*}\left( {X_{{B_{rj} }} - X_{{B_{ij} }} } \right) + r_{6} {*}\left( {X_{best} - X_{{B_{rj} }} } \right)$$

6 $$X_{{B2_{ij} }} = X_{{B_{ij} }} + { }r_{7} {*}\left( {X_{{B_{ij} }} - X_{{B_{rj} }} } \right) + r_{8} {*}\left( {X_{best} - X_{{B_{ij} }} } \right)$$

where $X_{best}$ demotes the best position provided by step B1, r5, r6, r7 and r8 denote random numbers between 0 and 1; $r$ and $i$ indicate two random agents, $\left\{ {r,i} \right\} \in \left\{ {1,2, \ldots ,NP} \right\}$.

**Algorithm 1:**
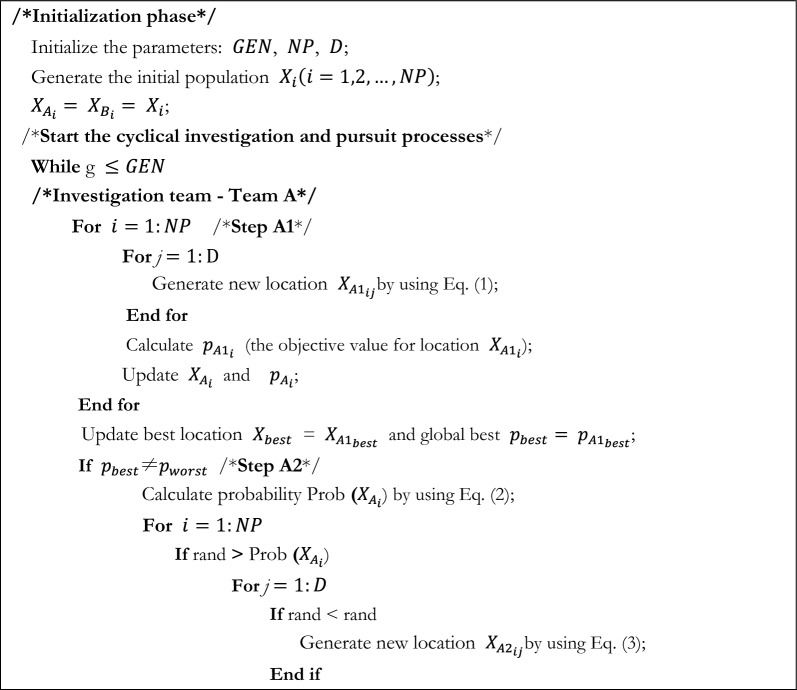

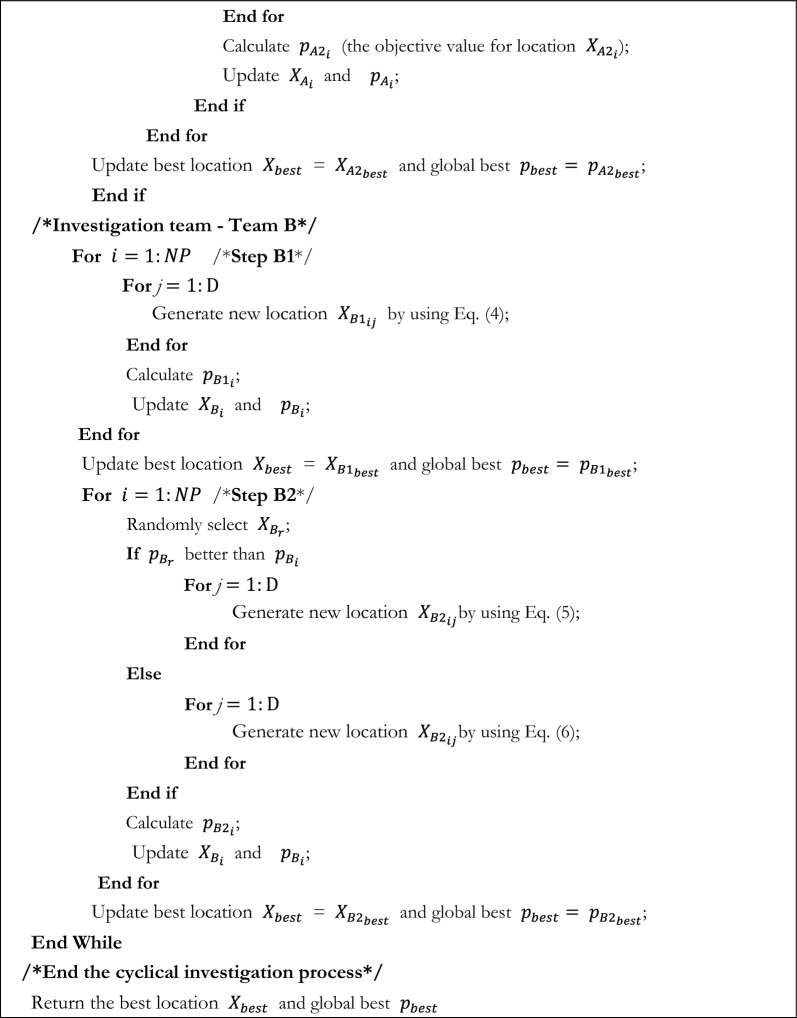
Pseudo-code of FBI.

### Slime mould algorithm (SMA)

Many newly developed algorithms based on their respective properties and search mechanisms can balance exploitation and exploration well. SMA is an effective population-based algorithm proposed by Li et al.^28^. SMA principally emulates the behavioral and morphological changes of slime moulds when they feed. The algorithm uses weights to model the positive and negative feedback slime moulds generate during the foraging process, resulting in three different morphological types. Previous studies in many application scenarios^53–56^ have demonstrated the superior performance of SMA in exploration and exploitation.

#### Approaching food

Slime moulds can approach food through odors in the environment. The following equation is used to model this contraction pattern of approaching food:

7 $$X\left( {t + 1} \right) = \left\{ {\begin{array}{*{20}l} {X_{b} \left( t \right) + vb\left( t \right) \cdot \left( {W \cdot X_{A} \left( t \right) - X_{B} \left( t \right)} \right),} \hfill & {r < p} \hfill \\ {vc\left( t \right) \cdot X\left( t \right),} \hfill & {r \ge p} \hfill \\ \end{array} } \right.$$

where $vb$ is a parameter between $- a$ and $a$, and $vc$ exhibits a linear decline from one to zero. $T$ represents the contemporary iterations, and $X_{b}$ indicates the position with utmost concentration of odor discovered thus far. $X$ refers the location of the slime moulds. $X_{A}$ and $X_{B}$ signify two fortuitously chosen individuals from the slime moulds. $W$ symbolizes the weights assigned to the slime moulds. $p$ is parameterized as follows:

8 $$p = {\text{tanh}}\left| {S\left( i \right) - DF} \right|$$

where $i \in 1,2, \ldots ,n,S\left( i \right)$ signifies the fitness evaluation of $X$, while $DF$ indicates the most superior fitness achieved across all iterations. $vb$ is given by the following equation:

9 $$vb = \left[ { - a,a} \right]$$

10 $${\text{a}} = {\text{arctanh}}\left( { - \left( {{\raise0.7ex\hbox{$t$} \!\mathord{\left/ {\vphantom {t {Max_{t} }}}\right.\kern-0pt} \!\lower0.7ex\hbox{${Max_{t} }$}}} \right) + 1} \right)$$

$W$ relies on the utilization of the following formula:

11 $$W\left( {SmellIndex\left( i \right)} \right) = \left\{ {\begin{array}{*{20}l} {1 + r \cdot \log \left( {\frac{bF - SmellOrder\left( i \right)}{{bF - wF}} + 1} \right),} \hfill & { condition} \hfill \\ {1 - r \cdot \log \left( {\frac{bF - SmellOrder\left( i \right)}{{bF - wF}} + 1} \right),} \hfill & { otherwise} \hfill \\ \end{array} } \right.$$

12 $$\left[ {SmellOrder,SmellIndex} \right] = sort\left( S \right)$$

where condition refers to when $S\left( i \right)$ is ranked within the superior half of the population, $r$ signifies a randomly selected value between 0 and 1. $max\_t$ represents the maximum iterations, and $bF$ and $wF$ refer the best fitness and worst fitness, respectively, achieved during the current iteration.$smellIndex$ indicates the sequence of fitness values for the ranking (which goes up in the minima problem).

#### Wrapping food

This section models the shrinking pattern of the venous tissue structure as the slime mould searches for food. As the concentration of food that the vein is subjected to increases, the intensity of the wave produced by the biological oscillator amplifies, the speed of cytoplasmic flow accelerates, and the thickness of the vein augments. Equation (13) provides a mathematical representation of the positive and negative feedback relationship between the width of the veins of the slime mould and the food concentration, where the parameter *r* models the uncertainty in the contraction pattern of the veins. Including a logarithmic function slows the change in frequency so that no drastic changes occur in contraction frequency values.$condition$ emulates the fact that the slime moulds dynamically tune their search pattern due to the concentration of food. In conditions of elevated food concentration, the weight of the neighborhood increases; vice versa, the weight of the vicinity decreases, prompting slime moulds to venture into alternative regions for exploration.

13 $$X\left( {t + 1} \right) = \left\{ {\begin{array}{*{20}l} {rand \cdot \left( {ub - lb} \right) + lb,} \hfill & {rand < z} \hfill \\ {X_{b} \left( t \right) + vb\left( t \right) \cdot \left( {W \cdot X_{A} \left( t \right) - X_{B} \left( t \right)} \right),} \hfill & {r < p} \hfill \\ {vc\left( t \right) \cdot X\left( t \right),} \hfill & {r \ge p} \hfill \\ \end{array} } \right.$$

where $lb$ and $ub$ denote the upper and lower boundary within a search range. $rand$ and $r$ represent random variables encompassing values inclusively between 0 and 1. $rand$ is used as a key parameter to control whether or not to enter the stochastic update, and $r$ determines whether or not to entry into the exploration and exploitation phase. Additionally, to adhere to the original text, the parameter $z$ is specifically assigned the value of 0.03.

#### Oscillation

Slime moulds rely heavily on propagating waves produced by a biological oscillator to manipulate the movement of cytoplasmic flow within the vein to a location that favors food concentration. To emulate the alterations in the pulse width of slime moulds, $W,vb,vc$ were used.

$W$ mathematically modeled the rate of oscillations of slime moulds relative to varying food concentrations. Consequently, this facilitated the slime moulds' ability to approach regions of higher food quality swiftly. In conditions of the food concentration was lower at certain locations, the slime moulds approached the food more slowly, thus improving the capability of the slime moulds to select the best food source efficiently.

**Figure 2:**
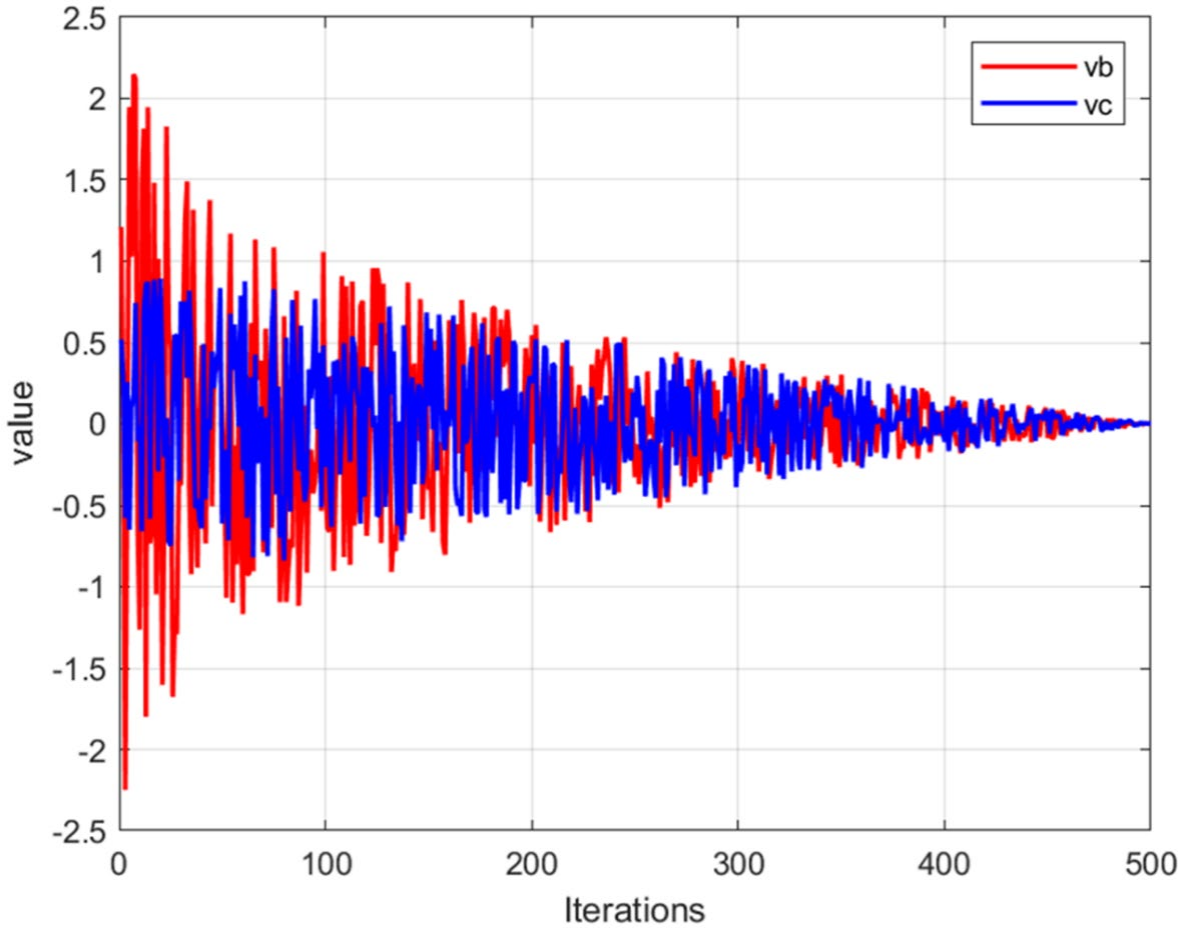
The trends of $vb$ and $vc$.

The variable $vb$ exhibits random oscillations between $\left[ { - a,a} \right]$, but steadily converges to 0 as the number of iterations increases. Similarly, the variable $vc$ undergoes oscillations within the interval of $\left[ { - 1,1} \right]$, and eventually converges to 0. The visual representation of this behavior can be observed in Fig. 2. The synergy between $vb$ and $vc$ simulates the selection patterns demonstrated by slime moulds. The slime moulds will explore some areas independently to find better food sources. The slime mould will branch out to search for better food sources instead of concentrating on one food source. This strategy ensures that the slimy bacteria algorithm will not easily fall into a local optimum.

## Proposed SMA_FBI

In this section, we provide a detailed description of the improved FBI and the time complexity of the algorithm in conjunction with the previous chapter.

### Enhanced FBI with the SMA (SMA_FBI)

Except for the population size and the evaluation of stopping conditions, the base FBI does not rely on predefined parameters, so the parameters do not affect the algorithm's behavior. At the beginning of the FBI algorithm, the population was replicated into two parts, and each part independently searched for the optimal solution, interacting information through the current optimal position and the best fitness value. However, this led to barriers to communication within the algorithm, and the two parts did not exchange other information. Kaveh et al.^47^ improved FBI by enhancing the exchange of information between the two parts. Instead of attempting to enhance communication, we added an inspector group to the investigating and searching groups, in which we introduced the search mechanism from the SMA.

When searching for food, the slime moulds can adaptively tune their search pattern due to varying levels of food concentration. In conditions of elevated food concentration, the slime moulds concentrate their search on the currently recognized food sources; if they find a low food concentration, the slime moulds depart from the food source and search for other food concentrations. Throughout this process, the front end of the slime mould extends out and is able to build a network of veins in the search space, and the quality of the food source affects the propensity of the slime mould to search.

**Figure 3:**
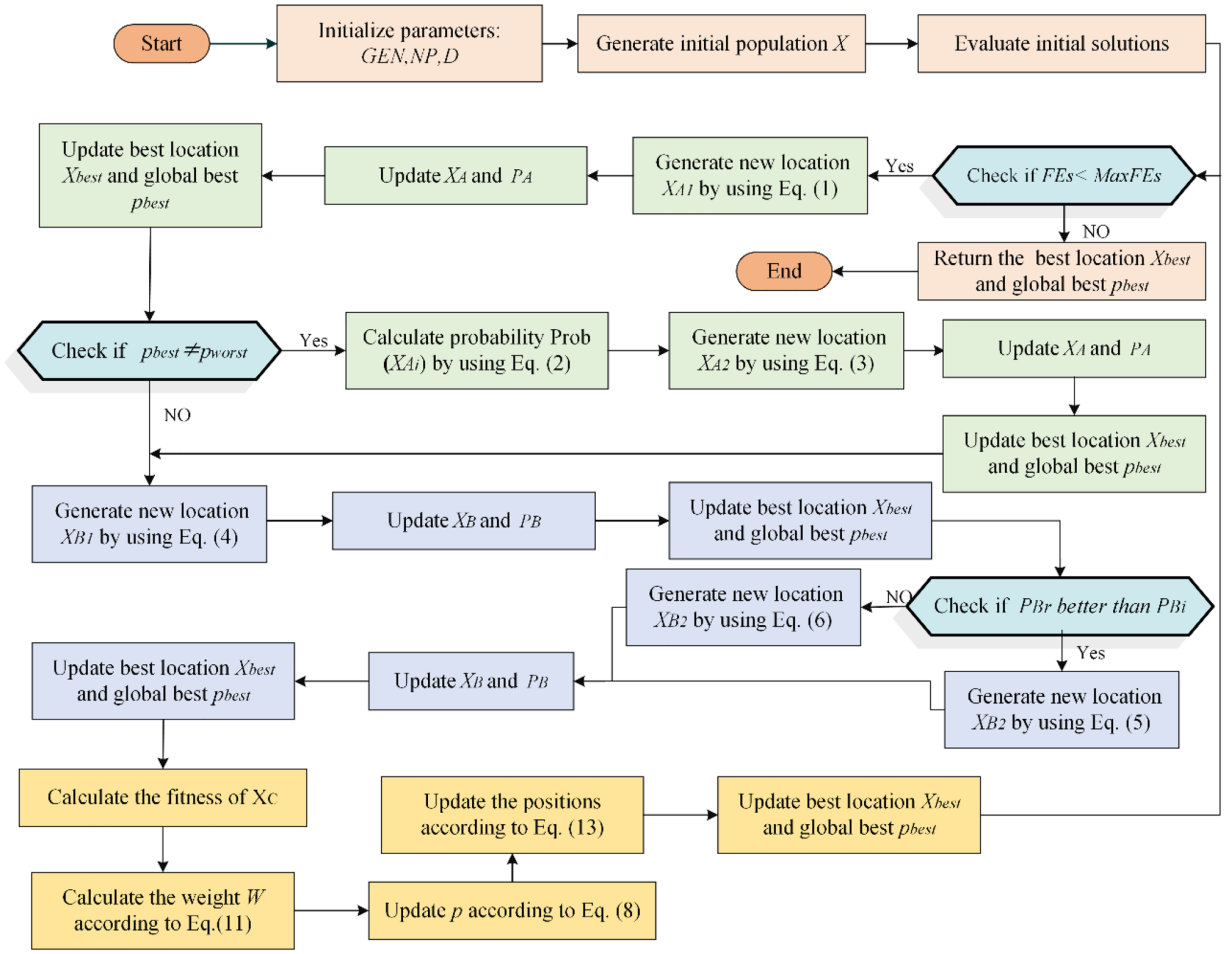
The flowchart of SMA_FBI.

Using this property of slime moulds in FBI, an inspector group can be reconstructed in addition to the original investigation group and pursuit group, which can make up for the deficiencies of the first two stages and enhance the capacity of the FBI. The flowchart of SMA_FBI is displayed in Fig. 3. The pseudo code for the SMA_FBI is provided by Algorithm 2.

**Algorithm 2:**
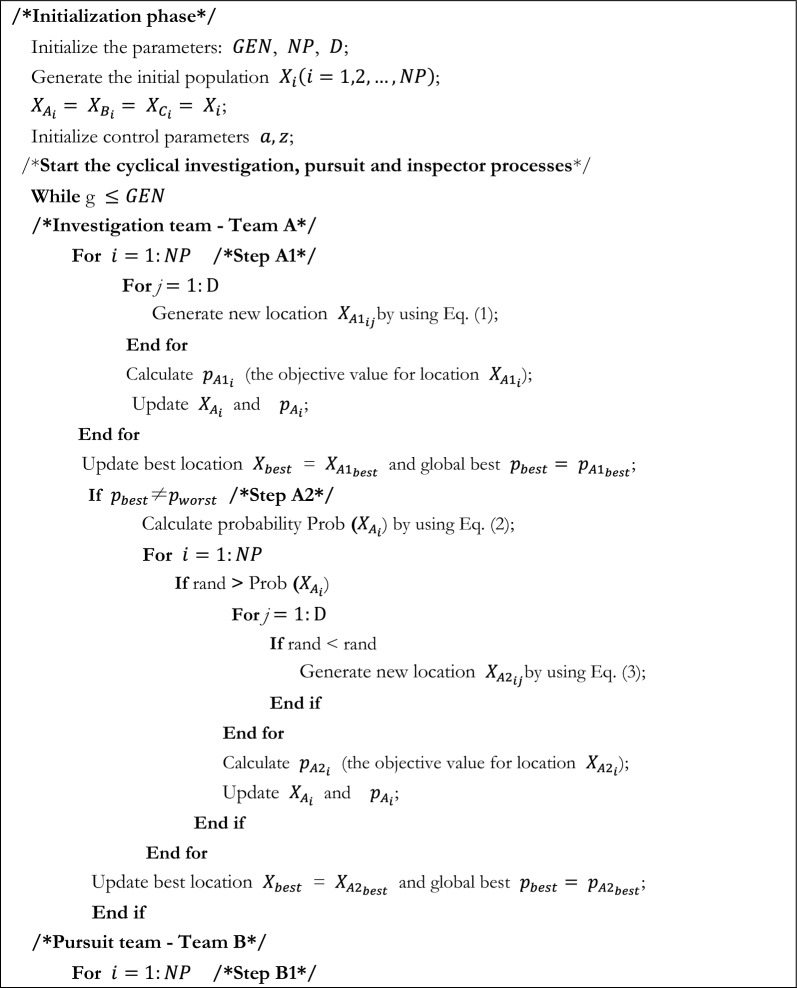

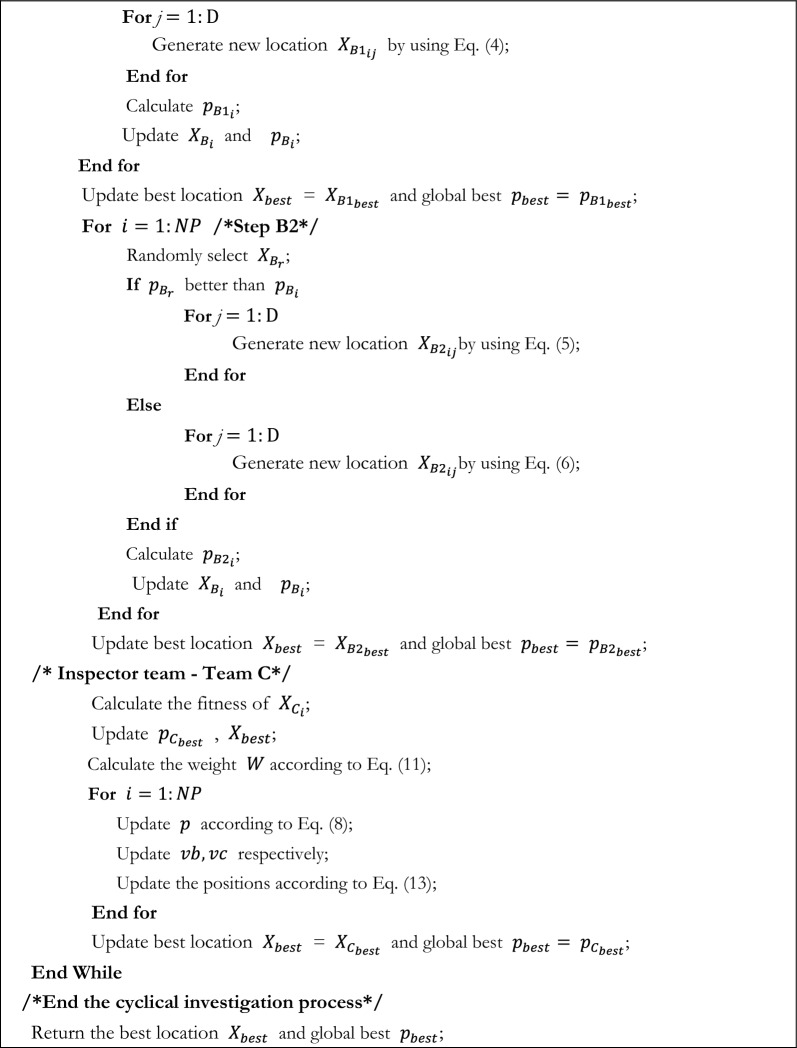
Pseudo-code of SMA_FBI.

### Computational complexity analysis

The time complexity of SMA_FBI is primarily associated with the dimension ($$D$$), the number of suspect locations ($$NP$$), and the number of evaluations ($$GEN$$). Overall, the time complexity is calculated from 4 aspects: initialization, fitness assessment, location update, and slime mould update strategy. For the initialization of the suspect location, the time complexity is $$O\left( {D*NP} \right)$$, the time cost of the adaptation assessment is $$O\left( {NP} \right)$$, the location updating part includes the investigation phase as well as the pursuit phase, and the time complexity of each phase is $$2* O\left( {NP*D} \right)$$, and the time consumption of the mucus updating strategy is $$O\left( {NP*\left( {1 + logN + D} \right)} \right)$$. Considering the total number of evaluations $$GEN$$, the total time complexity of SMA_FBI is $$O(D*NP + NP + GEN*\left( {4*NP*D + NP*\left( {1 + logN + D} \right)} \right)$$.

## Experiments

In order to assess the efficacy of the proposed algorithm SMA_FBI, a substantial quantity of experiments is undertaken in this section. Firstly, SMA_FBI is compared with 10 other original and 10 improved algorithms on 30 benchmark functions in CEC2017^57^. Benchmark datasets serve as widely acknowledged instruments for assessing the performance of various technologies against uniform criteria^58,59^. These datasets facilitate the evaluation of different technological dimensions, determining which technology excels over others across multiple domains^60,61^. Secondly, the algorithms are tested on different number of evaluations as well as different population sizes while controlling for other variables, and the complexity of SMA_FBI is also investigated and explained. Finally, the effectiveness of SMA_FBI in practical applications is tested on the GS dataset.

To emphasize the impartiality of the experiments, all comparison algorithms undergo testing within the same hardware environment. Within the continuous optimization experiments, metaheuristic algorithm parameters are configured with a population size set at 30, and a maximum of 300,000 assessments. At the same time, to mitigate the influence of randomness on the experiments, all the algorithms are repeated on the test function for 30 times. Based on the experimental data, the capability of the comparison algorithms was evaluated using the mean ($$avg.$$) and standard deviation ($$std.)$$ of the optimal function values. The best results in the data are shown in bold. The nonparametric statistical test Wilcoxon signed-rank test^62^ was utilized to ascertain whether SMA_FBI exhibits statistical superiority over other algorithms, with a significance level set at 0.05. The symbols " + / = /−" denote the proposed algorithm's superiority, equality, or inferiority to the other algorithms. Consistency was assessed using the Friedman test^63^ to rank the mean experimental results and list the average ranked value (ARV). Accurate validation of any proposed model or algorithm must be done based on known parameters and settings. In the next experiments, all the parameter settings of the compared algorithms will be listed separately.

The experimental results of SMA_FBI in terms of function optimization are shown and analyzed in this subsection. Thirty functions of CEC2017 are selected as test functions, and the specifics of these functions can be found in Appendix A.1. Within this selection, F1-F3 represent unimodal functions, F4-F10 are associated with multimodal functions, F11-F20 pertain to hybrid functions, and F21-F30 are linked to composite functions. The unimodal function contains a single global optimal solution, which serves as a means to assess the algorithm's exploitation capabilities. Meanwhile, the multimodal function has multiple locally optimal solutions and is employed to evaluate the algorithm's capacity for global exploration. The hybrid and composite functions gauge the algorithm's equilibrium between exploitation and exploration.

All assessments were conducted on a Windows Server 2012 R2 datacenter operating system equipped with 128 GB of memory, utilizing an Intel (R) Xeon (R) E5-2650 v4 (2.20 GHz) CPU, within a MATLAB R2014b programming environment.

In “Parameter sensitivity analysis” section, experiments are analyzed for different evaluation numbers as well as population size. “Comparison with conventional algorithms” and “Comparison with state of the art algorithms” sections entail comparisons of SMA_FBI with 10 original algorithms and 10 enhanced algorithms, respectively, aimed at substantiating SMA_FBI's performance in addressing exploration and exploitation in the context of CEC 2017. Furthermore, in “Experiments on real world optimization of GS” section, SMA_FBI is used to handle the GS problem for a dataset from the UCI database.

### Parameter sensitivity analysis

In order to enhance the analysis of the algorithm's parameter sensitivity, the impact of population size and the number of evaluations on the algorithm is examined by manipulating individual parameters while holding other variables constant.

During this phase of the experiment, in order to reflect the comprehensiveness of the experiment, four different functions, namely, unimodal function F3, multimodal function F7, hybrid function F14, and composite function F29, were selected for verification. The population sizes were set to 10, 30, 60, 100 and 200 to study the influence of population size on the algorithm's performance. Based on the findings presented in Appendix A.2, the optimization effectiveness of SMA_FBI generally surpasses that of FBI. Moreover, when the population size is 30, the optimization effect of SMA_FBI reaches the optimal value, while the optimization effect of SMA_FBI is relatively poor when the population size deviates from 30.

Another pivotal factor influencing the experimental results is the number of algorithm evaluations. We selected five evaluation times, 50,000, 100,000, 150,000, 200,000, and 300,000, to investigate the effect of evaluation times on the property of SMA_FBI. Similarly, the validation is carried out in four functions: unimodal function F3, multimodal function F7, hybrid function F14, and composite function F29. From Appendix A.3, it can be seen that SMA_FBI has achieved the optimal value on the composite function before 50,000 evaluations. For the multimodal and hybrid functions, the optimal value is already close to the optimal value at 200,000 evaluations, but only at 300,000 evaluations can all the functions take the optimal value. In conclusion, we chose 300,000 evaluations.

### Comparison with conventional algorithms

**Figure 4:**
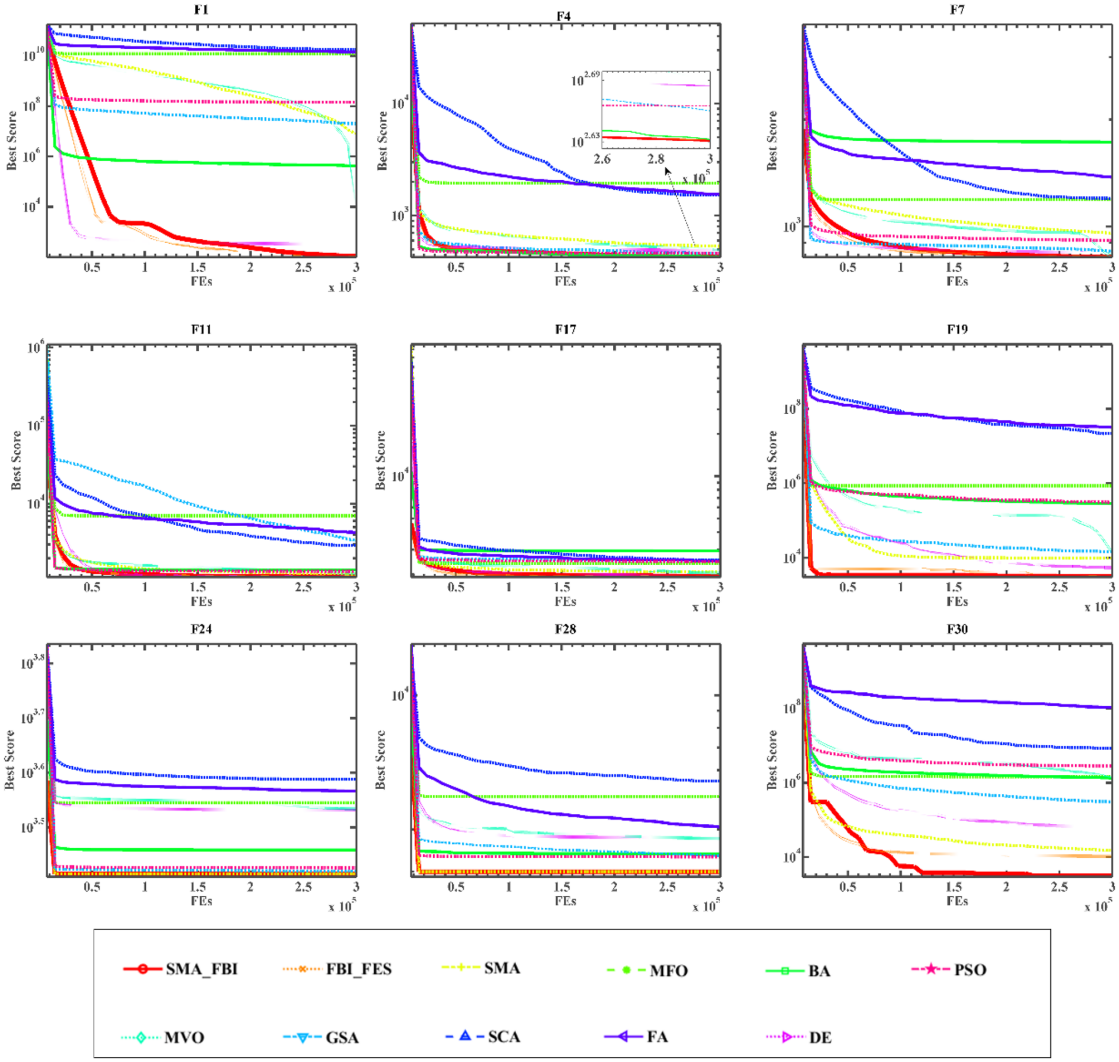
Convergence curves of SMA_FBI and ten conventional algorithms on nine functions.

Within this section, a comparison is conducted between SMA_FBI and ten different traditional algorithms, including the original FBI^41^ as well as SMA^28^, MFO^64^, BA^20^, MVO^65^, GSA^29^, SCA^25^, FA^66^, DE^19^, and PSO^67^. These ten algorithms used for comparison with the proposed algorithm include the original FBI as well as the SMA algorithm (for verifying the effectiveness of the improved algorithm), and also include the classical algorithms DE, PSO and different kinds of algorithms such as MVO, GSA, etc. (for verifying the superiority of the proposed algorithm). Table 1 showcases the precise parameter configurations employed by the comparison algorithms, with the specific parameter values remaining consistent with those used in the paper in which these algorithms were first presented. Appendix A.4, Appendix A.5 lists the detailed experimental data for the above algorithms on the F1-F30 test functions, and the algorithm convergence curves are shown in Fig. 4.

**Table 1:** The specific configuration of parameters.

Algorithms	Parameters and values
SMA	$z = 0.03$
MFO	$b = 1;t = \left[ { - 1,1\left] {;a \in } \right[ - 2, - 1} \right]$
BA	$a = 0.5;r = 0.5$
MVO	$WEO\_Max = 1;WEP\_Min = 0.2$
GSA	$G_{o} = 100;alpha = 20;final_{per} = 2$
SCA	$a = 2$
FA	$alpha = 0.5;beta = 0.2;gamma = 1$
DE	$beta\_min = 0.2;beta\_max = 0.8;pCR = 0.2$
PSO	$c1 = 2;c2 = 2;vMax = 6$

Appendix A.4, Appendix A.5 results show that SMA_FBI clearly outperforms the original algorithms, including FBI, SMA, and DE. The detailed data in Appendix A.4 shows that SMA_FBI performs best on 14 out of 30 functions and achieves suboptimal results on 8 functions, F1-2, F8, F11-12, F16-17, and F19. In the table, the optimal values are bolded. On the unimodal functions, SMA_FBI does not obtain the optimal values, but it also obtains the suboptimal values or better values, which shows the strong exploitation ability of SMA_FBI. Meanwhile, SMA_FBI obtains optimal values on most multimodal functions, which shows that SMA_FBI has strong global exploration ability. SMA_FBI is slightly larger than DE on F11, slightly larger than FBI on F12, slightly larger than DE on F16, and slightly larger than FBI on F17 and F19. Moreover, SMA_FBI obtains the optimal values on all the composite functions. This is due to the inclusion of the slime mould search mechanism, which improves the performance of FBI and ensures that the algorithm balances between exploration and exploitation within the search space.

The results of the Wilcoxon signed-rank test can be found in Appendix A.5. Observing the *p*-value from Appendix A.5 reveals that SMA_FBI demonstrates a significant improvement compared to the other MAs, with its *p*-value consistently lower than 0.05 for all algorithms except FBI and SMA. In addition, it can be seen that SMA_FBI also has some improvement effects for the FBI and SMA.

Based on the convergence curves of the 11 algorithms on 9 functions demonstrated in Fig. 4, we can observe that SMA_FBI achieves a high convergence rate on most of the functions, and in F1 as well as F30, the optimal solution is achieved although at a relatively gradual pace. This finding substantiates the claim that the introduction of the slime mould search mechanism, the added inspector group in addition to the original two search groups, enhances the performance of the FBI.

In summary, SMA_FBI demonstrates good overall superiority as well as robustness compared to other excellent original algorithms. Its comprehensive superiority and reliability are evident. Integrating the slime mould mechanism enhances the exploration and exploitation of the original FBI algorithm, leading to higher-quality solutions.

### Comparison with state-of-the-art algorithms

Within this experiment phase, the same CEC2017 benchmark function test set has been chosen to evaluate the capability of SMA_FBI in correlation to 10 state-of-the-art algorithms, namely EPSDE^68^, ALCPSO^69^, BMWOA^70^, CLPSO^71^, IGWO^72^, CESCA^73^, RDWOA^74^, LSHADE^75^, CBA^76^, and DECLS^77^. These 10 algorithms contain improved versions of various algorithms, especially of DE, PSO. EPSDE and LSHADE are two champion algorithms that have performed well in the field of evolutionary algorithms, and the superior performance of the proposed algorithm can be verified by comparing it with ten algorithms including these two. Table 2 shows the detailed parameter settings of the algorithms mentioned above. Appendix A.6 shows the comparison outcomes between SMA_FBI and the above advanced algorithms.

**Table 2:** The detailed parameter settings.

Algorithms	Parameters and values
EPSDE	$FF = \left[ {0.4,0.9\left] {;CR = } \right[0.1,0.9} \right]$
ALCPSO	$w = 0.4;c1 = c2;lifespan = 60;T = 2;pro = 1/n$
BMWOA	$a1 = \left[ {0,2} \right];a2 = \left[ { - 2, - 1} \right];b = 1$
CLPSO	$w \in \left[ {0.9,2} \right];c = 1.496;m = 5$
IGWO	$a \in \left[ {0,2} \right]$
CESCA	$a = 2$
RDWOA	$a1 = \left[ {0,2} \right];a2 = \left[ { - 2, - 1} \right];b = 1;s = 0$
LSHADE	$p_{best\_rate} = 0.11;arc_{rate} = 1.4;L_{Rate} = 0.8;First_{{class_{percentafe} }} = 0.5$
CBA	$Qmin = 0;Qmax = 2$
DECLS	$beta_{min} = 0.2;beta_{max} = 0.8;pCR = 0.2;m = 1500$

As can be noted from the summed rankings in Appendix A.6, SMA_FBI is still number one, even in the face of competition from the most highly acclaimed algorithms. In some of the previous functions of CEC2017, SMA_FBI did not achieve the best result compared to the champion algorithm, but it took the better solution. Furthermore, SMA_FBI achieves the optimal solution on most of the composite functions, i.e., F23, F25-30, and the sub-optimal solution on F24, and the std is 0 on all these functions, which indicates that SMA_FBI is more stable as well as robust on the composite functions. It shows that introducing the search mechanism of the SMA makes the algorithm more balanced between exploitation and exploration.

The results of the Wilcoxon signed-rank test, comparing SMA_FBI with other state-of-the-art algorithms, are depicted in Appendix A.7. From the table in Appendix, it can be observed that in the experiments of SMA_FBI with BMWOA, IGWO, CESCA, CBA, the *p*-value is much less than 0.05, which proves that SMA_FBI outperforms these algorithms. Meanwhile, compared with other algorithms, most are also less than 0.05, which shows that SMA_FBI has apparent advantages over them.

**Figure 5:**
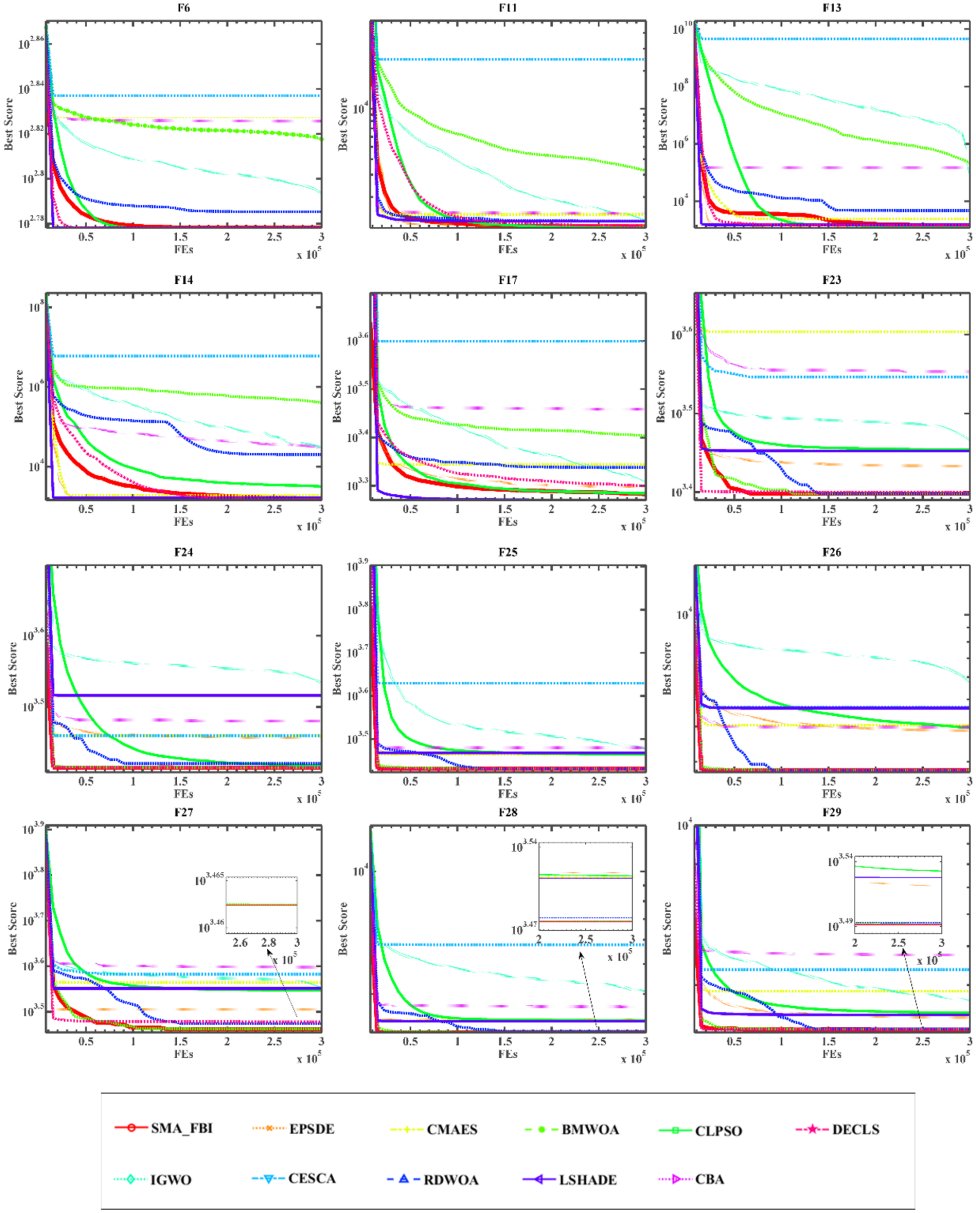
Convergence curves of SMA_FBI and ten state-of-the-art algorithms on twelve functions.

As shown in Fig. 5, SMA_FBI also shows competitive performance compared to the state-of-the-art and improved algorithms, proving that SMA_FBI is more competitive. The comparison algorithms also contain some improvements of DE and PSO algorithms, which further verifies that the introduction of the slime mould mechanism serves as a significant enhancement to the FBI.

In summary, in the face of competition from the challenging state-of-the-art algorithms, the optimization ability of SMA_FBI is reflected in the overall optimization performance in different types of functions, especially in composite and hybrid functions. The slime mould search mechanism, as the third search scheme in the improved algorithm, enhances the algorithm's exploration and search capability as a whole.

### Experiments on real-world optimization of GS

In this section, we employ the proposed algorithm SMA_FBI to address the GS problem and showcase the improved algorithm's effectiveness. Whereas the GS problem is a binary optimization task, we adapt the continuous SMA_FBI into a discrete variant, i.e., BSMA_FBI, to solve the high-dimensional GS problem.

#### Basic information

The GS problem requires selecting a set of most representative subsets from a collection of features for the purpose of dimensionality reduction of a dataset. GS can effectively reduce the computational cost of data, so many domains with large datasets wish to downsize application data.

In SMA_FBI based GS algorithm, $x = \left( {x_{i,1} , x_{i,2} , \ldots , x_{i,n} } \right)$ represents a set of features, if $x_{i,1} = 1$, it implies that the $i$ th feature is selected; otherwise, the feature is not selected. GS represents a discrete optimization problem; therefore, converting the SMA_FBI algorithm to a binary version is necessary. We utilize a transfer function to convert continuous SMA_FBI to binary SMA_FBI(BSMA_FBI). The machine learning algorithm is employed in a classification capacity, and its classification accuracy is utilized to evaluate the ability of BSMA_FBI to screen important features in the dataset. In addition, during the evaluation process, cross-validation was employed to assess the optimum subset of features used for classification to avoid the impact of random elements on the experiment.

#### Fitness function and implementation of experiments

In previous work on continuous optimization, the proposed SMA_FBI searches for optimal solutions in a continuous search space. Whereas the GS problem is a binary problem, such a problem requires that the solution must be binary, i.e., it can only take either 0 or 1. However, many optimization algorithms are inherently designed for continuous spaces. Therefore, we need a way to convert the outputs of these continuous optimizers to binary values to satisfy the requirements of the problem. The transfer function (or threshold function) is the key to this conversion. The basic idea is to set a threshold value for the output of the continuous optimizer, and then convert the output to 0 or 1 according to this threshold value, with 1 indicating selected and 0 indicating unselected. By adjusting the threshold, we can control the stringency of the selected genes. A higher threshold will result in fewer genes being selected, while a lower threshold may result in more genes being selected. Here we choose a threshold of 0.5 as shown below:

14 $$X_{i}^{j} = \left\{ {\begin{array}{*{20}l} 0 \hfill & {rand \le 0.5} \hfill \\ 1 \hfill & {rand > 0.5} \hfill \\ \end{array} } \right.$$

$X_{i}^{j}$ is the $i$ th value of the current search entity in the $j$ th dimension within the discrete space.

The transfer function is a proper translator that converts a continuous optimization algorithm into a discrete variant of the algorithm without altering the structure of the algorithm, which is convenient and efficient. Within this paper, $V$- type transfer function is employed, and its transfer function is shown below:

15 $$T\left( {x_{i}^{j} \left( t \right)} \right) = \left| {\tanh x_{i}^{j} \left( t \right)} \right|$$

GS is a process of obtaining the lowest classification error rate employing the least subset of features, which needs to be achieved simultaneously. Evidently, the GS problem presents itself as a multi-objective optimization challenge, and to satisfy each objective, a fitness function can be designed using the classification error rate and the number of selected features to evaluate the chosen feature subset. The specific form of the fitness function is shown below:

16 $$fitness = { }a \cdot error + { }b \cdot \frac{l}{d}$$

where $error$ represents the classification error rate computed by the K-Nearest Neighbor (KNN)^78^ classifier, $l$ signifies the size of the selected feature subset, and $d$ is the total number of features in the dataset. Meanwhile, $a$ and $b$ serve as two weighting factors indicating the significance of the classification error and the subset length, respectively, to the GS problem. Our study asserts that the classification error rate deserves more attention than the feature subset length. Thus, we assign $a$ to be 0.95 and $b$ to be $1 - a$, i.e., 0.05. Each feature subset is evaluated based on fitness, with smaller fitness values indicating superior feature subsets.

#### Experimental results and analysis of FS

The SMA_FBI based GS method, which we refer to as BSMA_FBI, will be in the face of competition from several state-of-the-art GS methods on 18 datasets, including bGWO^79^, BBA^80^, BGSA^81^, BPSO^82^, bALO^83^, BSSA^84^, bHHO^85^, and the original GS algorithm for the FBI, BFBI. These algorithms used for comparison are more classical algorithms and are commonly used in comparison experiments. They include many different kinds of algorithms such as nature-inspired algorithms, algorithms inspired by physical phenomena, and so on. Table 3 lists the detailed parameters of these classifiers. GS based on the SMA_FBI algorithm is performed on each dataset and is run $N$ times, and tenfold cross-validations are performed each time. The data samples are partitioned into training, validation, and test sets in the cross-validation procedure according to a certain ratio. In this paper, the KNN classifier is used for classification. The classifier initially undergoes training and classification on all the data within the training set, subsequently assessing and validating against the samples in the validation set, and ultimately applies the chosen features to the test data to ascertain computational accuracy.

**Table 3:** Parameter settings for the classifiers.

Algorithms	Parameters and values
bGWO	$a = \left[ {0,2} \right]$
BBA	$a = 0.5;r = 0.5$
BGSA	$wMax = 20;wMin = 1e - 10$
BPSO	$Max = 0.9;Min = 0.4$
BSSA	$a = 2$
bHHO	$a = \left[ {0,2} \right]$

Table 4 lists 18 detailed features from the UCI dataset, including the number of instances, features, and categories. As can be observed from the table, these datasets have 32–6598 samples, 23–15,010 features, and 2–26 classes. These datasets essentially represent different types of data, containing both small high-dimensional samples and large low-dimensional samples, which challenges the performance of the algorithm.

**Table 4:** Characteristics of gene expression datasets.

Datasets	Samples	Features	Categories	Datasets	Samples	Features	Categories
Parkinson	195	23	2	Leukemia1	72	5328	5
penglungEW	102	10,510	2	Leukemia2	72	11,226	3
clean2	6598	167	2	Lung_Cancer	203	12,601	3
Colon	62	2000	2	Lungcancer_3class	32	57	3
Leukemia	72	7131	2	Prostate_Tumor	102	10,509	2
Brain_Tumor1	90	5920	5	SRBCT	83	2309	4
Brain_Tumor2	50	10,368	4	Tumors_9	60	5726	9
CNS	60	7130	2	Tumors_11	174	12,534	11
DLBCL	77	5470	2	Tumors_14	308	15,010	26

Appendix A.8–Appendix A.11 reflects the statistical findings of the means by the number of features selected, error rates, fitness values, and computation time. The bolded values represent the most favorable outcomes for the present comparison results. Examination of Appendix A.8 distinctly illustrates that the proposed BSMA_FBI selects the fewest features across nearly all datasets and achieves the second least number of features on the Parkinson, Lungcancer_3class dataset. By comparing the data of BSMA_FBI with BFBI, we can also find that our improvement of FBI is very effective, and our proposed algorithm selects fewer features and fetches better results than the original algorithm. The ARV metric shows the ranking results of various algorithms on multiple datasets, and there is no doubt that BSMA_FBI is ranked first. This shows that BSMA_FBI is competitive in selecting the least features.

According to the ARV, comparison results in Appendix A.9, as a whole, BSMA_FBI has not achieved the optimal results, but it has also achieved a suboptimal ranking, with an average error that is only a little bit higher than that of bGWO, and the average error value of BSMA_FBI is noteworthy lower when compared with that of BFBI. The proposed algorithm achieves the least average error on more than half of the datasets and has the smallest standard deviation, even though many of them are 0. This indicates that the proposed algorithm is very stable, which also proves the algorithm's superior behavior. Of course, we can also see that BSMA_FBI achieves relatively poor results on some algorithms, especially Tumors_9, Tumors_11, and Tumors_14, and we speculate that it may be the fact that these three datasets contain too many categories, which leads to the algorithm's general effect.

Appendix A.10 demonstrates the fitness values for the algorithm comparison, i.e., the weighted results of the error rate versus the number of features, from which it is evident that the data is mainly in line with the trend in Appendix A.9, although BSMA_FBI achieves the best results due to the addition of the number of features as a factor. The ARV results show that BSMA_FBI outperforms bGWO, and both significantly outperform the other optimizers, with BFBI having the worst results. This proves that the entry of the slime mould algorithm as a mechanism that improves the effectiveness of the original algorithm in searching for suitable features in the feature space has a positive impact.

Based on the average computation time results in Appendix A.11, it can be observed that although the BSMA_FBI algorithm has a high computation time, it is still superior to BFBI, which can also prove the value of the improvement side by side, reducing the time cost.

Tables 5 and 6 show the Wilcoxon signed-rank test results of BSMA_FBI against other GS optimizers in terms of classification error as well as the number of features selected, respectively. From Table 5, it can be seen that there seems to be no significant difference between BSMA_FBI and other GS optimizers in terms of classification error and only in a few tests is the *p*-value less than 0.05. However, it can be seen from Table 6 that there is a significant difference between BSMA_FBI and other gene selection optimizers in terms of the number of features selected. This indicates that SMA_FBI has a significant advantage over these algorithms.

**Table 5:** The *p*-value of the Wilcoxon test between the BSMA_FBI and alternative GS optimizers on average error rate.

	bGWO	BBA	BGSA	BPSO	bALO	BSSA	bHHO	BFBI
Brain_Tumor1	1.00E+00	1.56E−02	4.38E−01	1.25E−01	1.25E−01	1.25E−01	2.50E−01	9.38E−02
Brain_Tumor2	5.00E−01	6.25E−02	2.50E−01	6.25E−02	1.25E−01	1.25E−01	2.50E−01	1.56E−02
clean2	1.95E−03	7.81E−03	1.95E−03	1.95E−02	1.95E−03	4.26E−01	4.75E−01	4.77E−01
CNS	1.00E+00	3.91E−03	2.50E−01	6.25E−02	3.13E−02	3.13E−02	6.25E−02	1.56E−02
Colon	3.75E−01	1.95E−03	3.13E−02	1.56E−02	7.81E−03	4.69E−02	1.56E−02	3.91E−02
DLBCL	1.00E+00	1.25E−01	1.00E+00	1.00E+00	1.00E+00	5.00E−01	1.00E+00	1.00E+00
Leukemia	1.00E+00	6.25E−02	1.00E+00	1.00E+00	1.00E+00	1.00E+00	1.00E+00	5.00E−01
Leukemia1	1.00E+00	3.13E−02	1.00E+00	1.00E+00	1.00E+00	1.00E+00	1.00E+00	1.00E+00
Leukemia2	1.00E+00	1.25E−01	5.00E−01	5.00E−01	1.00E+00	1.00E+00	1.00E+00	5.00E−01
Lung_Cancer	6.25E−01	3.91E−03	5.31E−01	3.28E−01	7.50E−01	3.13E−01	1.25E−01	1.56E−02
lungcancer_3class	1.00E+00	1.56E−02	1.00E+00	1.00E+00	1.00E+00	1.00E+00	1.00E+00	1.00E+00
Parkinson	2.03E−01	1.56E−02	7.42E−01	6.25E−02	3.13E−02	1.72E−01	3.59E−01	6.72E−01
penglungEW	1.00E+00	3.13E−02	5.00E−01	1.00E+00	5.00E−01	5.00E−01	1.00E+00	3.13E−02
Prostate_Tumor	1.00E+00	1.95E−03	5.00E−01	1.25E−01	2.50E−01	2.50E−01	5.00E−01	3.13E−02
SRBCT	1.00E+00	3.13E−02	1.00E+00	1.00E+00	1.00E+00	1.00E+00	1.00E+00	1.00E+00
Tumors_11	1.41E−01	2.54E−02	5.47E−01	2.19E−01	8.44E−01	1.00E+00	7.62E−01	3.20E−01
Tumors_14	1.95E−03	1.31E−01	9.77E−03	7.70E−01	1.05E−01	9.22E−01	4.96E−01	1.05E−01
Tumors_9	1.00E+00	7.81E−03	1.88E−01	7.50E−01	5.00E−01	3.75E−01	5.00E−01	3.13E−02

**Table 6:** The *p*-value of Wilcoxon test between the BSMA_FBI and alternative GS optimizers on average number of the selected features.

	bGWO	BBA	BGSA	BPSO	bALO	BSSA	bHHO	BFBI
Brain_Tumor1	1.95E−03	1.95E−03	1.95E−03	1.95E−03	1.95E−03	1.95E−03	1.95E−03	1.95E−03
Brain_Tumor2	1.95E−03	1.95E−03	1.95E−03	1.95E−03	1.95E−03	1.95E−03	1.95E−03	1.95E−03
clean2	2.03E−01	1.95E−03	1.95E−03	1.95E−03	1.95E−03	2.73E−02	3.91E−03	1.95E−03
CNS	1.95E−03	1.95E−03	1.95E−03	1.95E−03	1.95E−03	1.95E−03	1.95E−03	1.95E−03
Colon	1.95E−03	1.95E−03	1.95E−03	1.95E−03	1.95E−03	1.95E−03	1.95E−03	1.95E−03
DLBCL	1.95E−03	1.95E−03	1.95E−03	1.95E−03	1.95E−03	1.95E−03	1.95E−03	1.95E−03
Leukemia	1.95E−03	1.95E−03	1.95E−03	1.95E−03	1.95E−03	1.95E−03	1.95E−03	1.95E−03
Leukemia1	1.95E−03	1.95E−03	1.95E−03	1.95E−03	1.95E−03	1.95E−03	1.95E−03	1.95E−03
Leukemia2	1.95E−03	1.95E−03	1.95E−03	1.95E−03	1.95E−03	3.91E−03	1.95E−03	1.95E−03
Lung_Cancer	1.95E−03	1.95E−03	1.95E−03	1.95E−03	1.95E−03	1.95E−03	1.95E−03	1.95E−03
lungcancer_3class	2.81E−01	1.95E−03	3.13E−02	1.95E−03	1.95E−03	1.95E−03	3.91E−03	1.95E−03
Parkinson	3.59E−01	1.95E−03	1.00E+00	4.06E−01	8.44E−01	1.95E−03	1.48E−01	1.95E−03
penglungEW	1.19E−01	1.95E−03	1.95E−03	1.95E−03	1.95E−03	7.81E−03	1.95E−03	1.95E−03
Prostate_Tumor	1.95E−03	1.95E−03	1.95E−03	1.95E−03	1.95E−03	1.95E−03	1.95E−03	1.95E−03
SRBCT	1.95E−03	1.95E−03	1.95E−03	1.95E−03	1.95E−03	1.95E−03	1.95E−03	1.95E−03
Tumors_11	1.95E−03	1.95E−03	1.95E−03	1.95E−03	1.95E−03	1.95E−03	1.95E−03	1.95E−03
Tumors_14	2.73E−02	1.95E−03	1.95E−03	1.95E−03	1.95E−03	3.91E−03	1.95E−03	1.95E−03
Tumors_9	8.40E−02	1.95E−03	1.95E−03	1.95E−03	1.95E−03	3.91E−03	9.77E−03	1.95E−03

**Figure 6:**
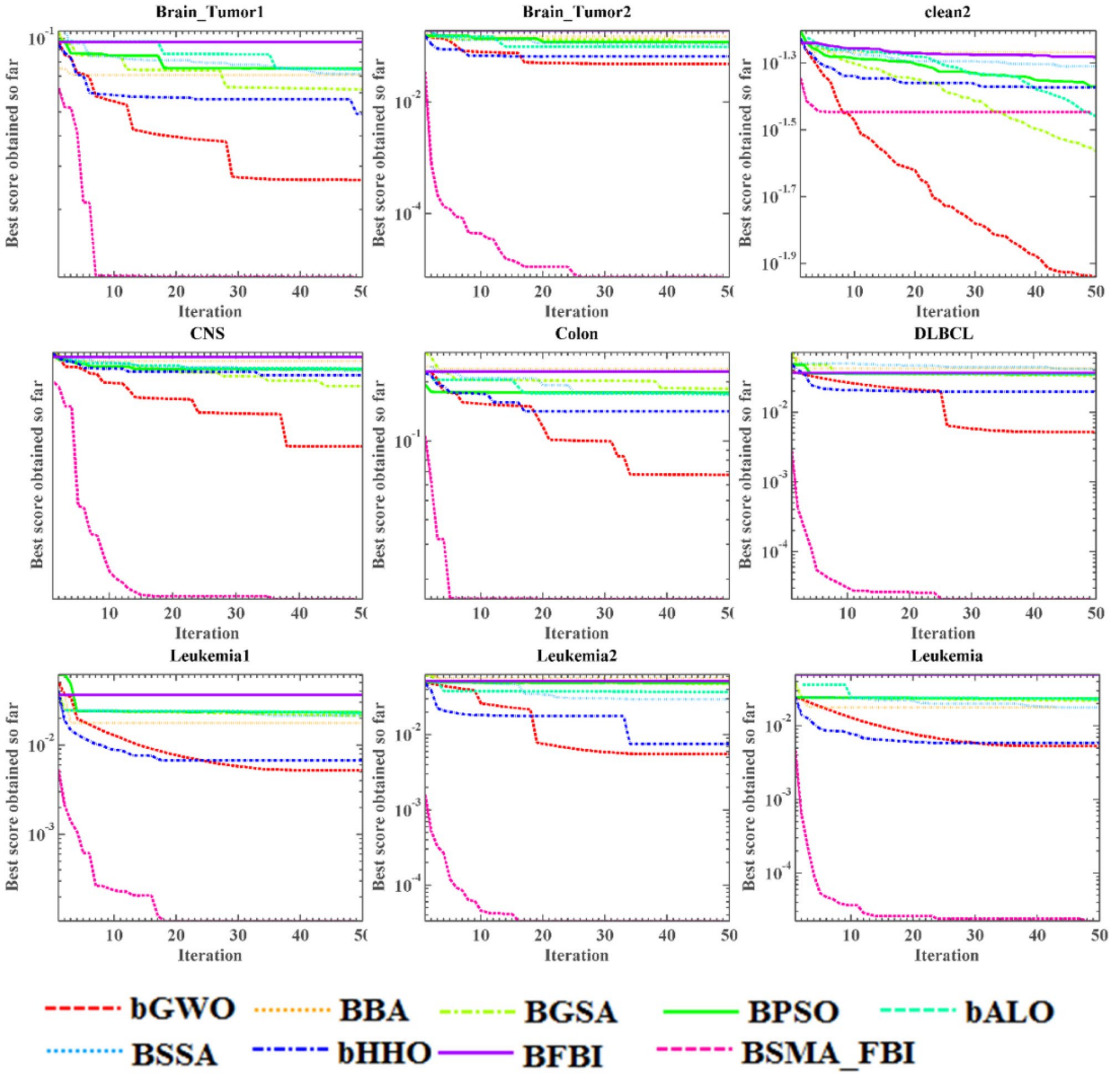
Convergence plots for the BSMA_FBI and alternative binary metaheuristic algorithms across 9 datasets.

**Figure 7:**
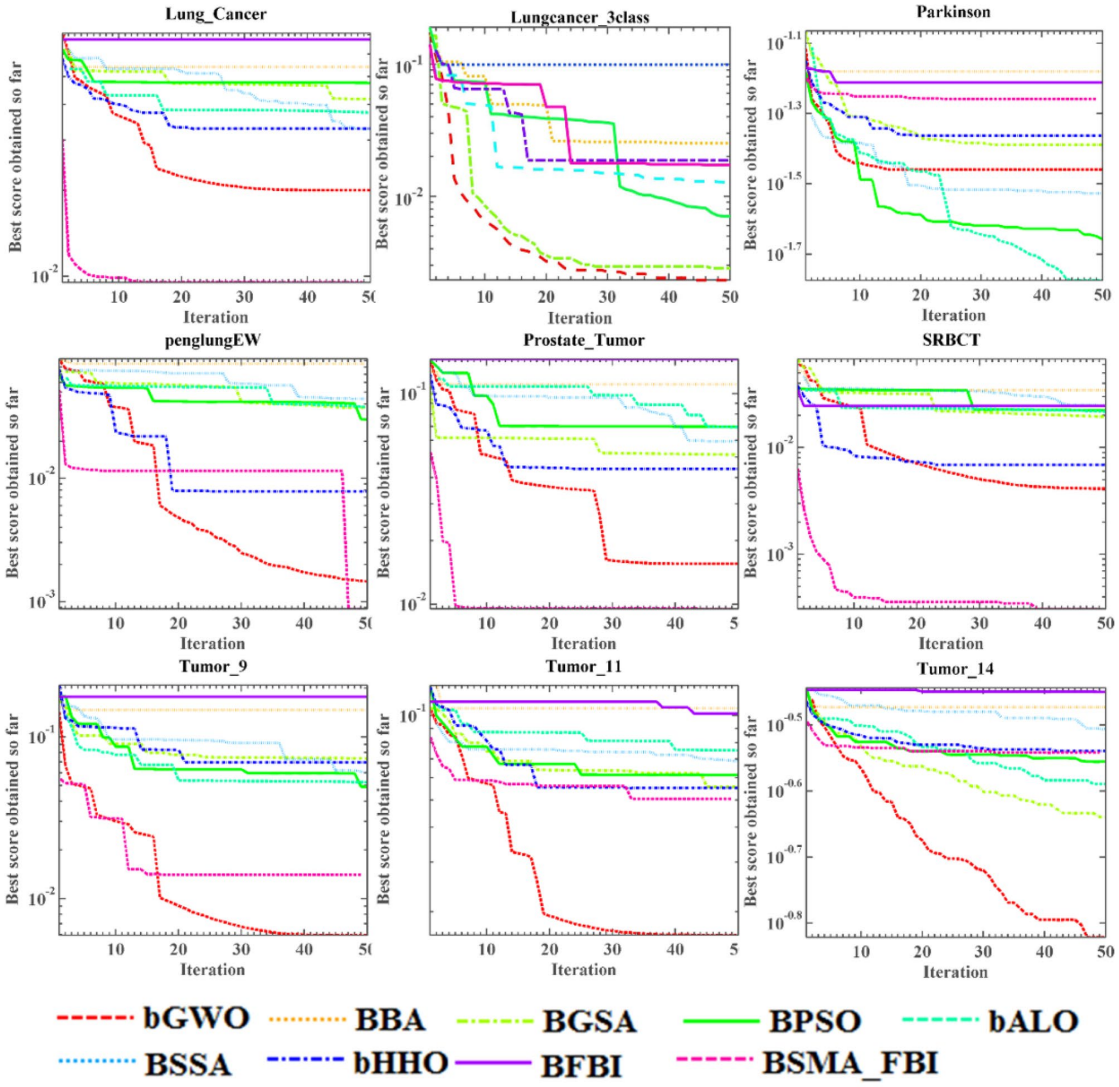
Convergence plots for the BSMA_FBI and alternative binary metaheuristic algorithms across 9 datasets.

The images are more intuitive and visual than the data in the tables. Figures 6 and 7 show the optimal fitness values calculated by multiple algorithms during the optimization process in the form of curves. The horizontal axis represents the iterations of the algorithms, and the vertical axis represents the average fitness value for every 10 runs of the nine algorithms. It can be seen that the best fitness values achieved by BSMA_FBI are much smaller than those of the other algorithms on most of the datasets, such as the Brain_Tumor1, Brain_Tumor2, and CNS datasets. Of course, on some datasets, the fitness values obtained by BSMA_FBI are relatively average, mainly due to trapping in the local optimum, such as the clean2, Lungcancer_3class datasets, etc. The BSMA_FBI algorithm's performance on the penglungEW dataset is relatively bright, and in the early stage of iteration, the fitness values obtained have been in the local optimum. Nevertheless, at the last moment of the iteration, it suddenly escapes from local optimality. It finds a better solution, which can be observed that introducing the slime mold algorithm as a mechanism enhances the diversity of the algorithm during the development period and the possibility of escaping from the local optimum.

## Discussion

This section summarizes the experimental findings of the proposed SMA_FBI on continuous function optimization and GS problems and provides a detailed analysis of the nature of the algorithms involved as well as the experimental results. The experimental part in “Experiments” section can be divided into three aspects: (1) Comparative experiments on the population size and on the number of algorithm evaluations in function optimization to find the most suitable population size and the number of evaluations; (2) On the CEC 2017 dataset, the correctness of the introduction of the slime mould search mechanism for the FBI is verified by comparing it to the base algorithm and state-of-the-art algorithms as well as the overall SMA_FBI algorithm's superiority. (3) SMA_FBI is applied to a high-dimensional GS optimization problem from UCI data to prove the algorithm's behavior for effective dimensionality reduction of high-dimensional data and addressing discrete combinatorial optimization challenges.

From the perspective of function optimization, it can be seen from Appendix A.4, Appendix A.5 as well as Fig. 4, that the algorithm after the integration of the slime mould mechanism is superior compared to the FBI, and its optimization ability is stronger. Secondly, the SMA_FBI algorithm can occupy an obvious advantage, no matter it is compared with the classical DE, PSO, or the novel SMA. In addition, according to the data in Appendix A.6, Appendix A.7 and the curves in Fig. 5, we can see the comparison between SMA_FBI and a variety of improved algorithms, which contain several champion algorithms (EPSDE, LSHADE), as well as other state-of-the-art and improved algorithms (e.g., ALCPSO, DECLS), and so on. We can see that SMA_FBI significantly outperforms these state-of-the-art algorithms. At the same time, we can also see that SMA_FBI is not optimal in some problems, especially in the hybrid function, which is not obvious when comparing with the basic algorithms, but when comparing with the state-of-the-art algorithms, this problem becomes evidently apparent.

In discrete combinatorial optimization, SMA_FBI achieves satisfactory results for GS problems. We evaluated BSMA_FBI (the binary version of the algorithm) as well as several GS optimizers using 18 datasets from the UCI repository (containing different types of data). Appendix A.8–Appendix A.11 quantitatively analyzes the performance of the algorithms in the four aspects of the number of selected features, classification errors, fitness values, and time cost, respectively. It is readily apparent that BSMA_FBI surpasses the other optimization techniques, and the proposed algorithm maintains high classification accuracy while selecting fewer features. It can also be seen that BFBI and BSMA_FBI algorithms are ranked in the bottom two positions in terms of time cost, while bGWO is also effective and has a higher time complexity. Given the significant reduction in time cost compared to the original algorithm, it is justifiable to take satisfaction in the performance of BSMA_FBI. In addition, we can see that BSMA_FBI is more effective on high-dimensional small-sample data but less effective on low-dimensional large-sample datasets and multi-classified data, which is the direction of our future improvement. In addition, Figs. 6 and 7 show that BSMA_FBI exhibits elevated classification accuracy and convergence at a superior rate compared to its counterparts. Thus, it shows that BSMA_FBI is a promising approach for discrete combinatorial optimization challenges in GS.

In brief, this article discusses the SMA_FBI algorithm, which incorporates a slime mould search mechanism based on the original FBI to achieve improved algorithm performance. By comparing with other excellent algorithms on the function optimization problem, it is found that SMA_FBI has a significant advantage in enhancing population diversity as well as convergence. In addition, compared with other GS methods on the GS problem, it is verified that BSMA_FBI can obtain higher classification accuracy while selecting fewer features. Of course, there is also a problem that BSMA_FBI has high time loss when performing GS, which is an optimization direction we need to consider afterward. Overall, SMA_FBI shows good prospects in addressing diverse optimization and GS problems.

Improving the accuracy and efficiency of gene selection plays a crucial role in medical diagnosis and personalized therapy, and has a profound impact on the development of drug discovery and individualized treatment. By improving the accuracy of gene selection, we are able to more accurately identify genetic variants associated with diseases and thus diagnose them more accurately. This helps to avoid misdiagnosis and underdiagnosis, and provide patients with more precise and personalized treatment plans. Meanwhile, in the process of drug development, by accurately selecting relevant genes, we can study the mechanism of action of drugs in greater depth, accelerate the process of drug development, reduce the cost of research and development, and improve the efficiency of research and development, so as to assist in medical diagnosis and personalized treatment.

## Conclusion and future work

Throughout this manuscript, we propose a modification of FBI, SMA_FBI, which significantly improves the capability of the original FBI. Based on the original two phases of the FBI, the slime mould search was introduced as the third phase to balance the equilibrium between exploitation and exploration better. Comparisons are made with multiple optimization algorithms on 30 CEC2017 datasets and 18 datasets from the UCI repository to validate the improvement and effectiveness of the algorithms.

The slime mould search mechanism adjusts its search pattern according to the current situation, which, in the early stage, helps to explore the search space quickly, while at a later stage, it helps to maintain the diversity of the population when developing of search space, thus avoiding falling into local optimality. Introducing the search mechanism in the FBI is equivalent to adding an inspector group to the original two investigation and search groups, significantly improving the algorithm's capability. This can be demonstrated by the experimental data given in “Experiments” section.

The SMA_FBI algorithm achieves superior behavior in continuous optimization tasks. In 4.2 and 4.3, SMA_FBI is compared with the original algorithms (DE, PSO, MFO, SMA, etc.) as well as the improved algorithms (EPSDE, LSHADE, ALCPSO, etc.), respectively, and is tested on the CEC2017 dataset, and the results show that SMA_FBI possesses strong global search capability as well as fast convergence speed. Also, the proposed algorithm performs satisfactorily in high-dimensional genetic data GS tasks. In 4.4, the binary version of the suggested algorithm is contrasted with various algorithms on 18 well-known datasets from the UCI database, showing that the algorithm can achieve high classification accuracy while selecting fewer features.

In conclusion, the SMA_FBI proposed in this paper provides excellent solutions to continuous optimization and discrete combinatorial optimization problems, encouraging the application of the algorithm to more complex and realistic challenges.

### Ethical statement

The manuscript has not be submitted to more than one journal for simultaneous consideration and has not been published elsewhere in any form or language.

### Supplementary Information

Supplementary Information.

## Data Availability

The data involved in this study are all public data, which can be downloaded through https://github.com/younyhoney/SMA_FBI/tree/main.
