# Supplementary material for: Advancing forensic-based investigation incorporating slime mould search for gene selection of high-dimensional genetic data
Source: Sci Rep. 2024 Apr 13;14:8599. doi: 10.1038/s41598-024-59064-w (PMC11016116; doi:10.1038/s41598-024-59064-w)
Supplement: Supplementary file 1 — Supplementary Information. [file 41598_2024_59064_MOESM1_ESM.docx]

Appendix A

**Appendix A.1.** Descriptions of CEC2017 functions. (Search range: [−100, 100] ^D^).

| **ID** | **Name of the function** | **Class** | **Optimum** |
| --- | --- | --- | --- |
|  |  |  |  |
| **C1** | Shifted and Rotated Bent Cigar Function | Unimodal | 100 |
| **C2** | Shifted and Rotated Sum of Different Power Function |  | 200 |
| **C3** | Shifted and Rotated Zakharov Function |  | 300 |
| **C4** | Shifted and Rotated Rosenbrock’s Function | Multimodal | 400 |
| **C5** | Shifted and Rotated Rastrigin’s Function |  | 500 |
| **C6** | Shifted and Rotated Expanded Scaffer’s F6 Function |  | 600 |
| **C7** | Shifted and Rotated Lunacek Bi-Rastrigin Function |  | 700 |
| **C8** | Shifted and Rotated Non-Continuous Rastrigin’s Function |  | 800 |
| **C9** | Shifted and Rotated Lévy Function |  | 900 |
| **C10** | Shifted and Rotated Schwefel’s Function |  | 1000 |
| **C11** | Function 1: N=3 | Hybrid | 1100 |
| **C12** | Function 2: N=3 |  | 1200 |
| **C13** | Function 3: N=3 |  | 1300 |
| **C14** | Function 4: N=4 |  | 1400 |
| **C15** | Function 5: N=4 |  | 1500 |
| **C16** | Function 6: N=4 |  | 1600 |
| **C17** | Function 6: N=5 |  | 1700 |
| **C18** | Function 6: N=5 |  | 1800 |
| **C19** | Function 6: N=5 |  | 1900 |
| **C20** | Function 6: N=6 |  | 2000 |
| **C21** | Function 1: N=3 | Composition | 2100 |
| **C22** | Function 2: N=3 |  | 2200 |
| **C23** | Function 3: N=4 |  | 2300 |
| **C24** | Function 4: N=4 |  | 2400 |
| **C25** | Function 5: N=5 |  | 2500 |
| **C26** | Function 6: N=5 |  | 2600 |
| **C27** | Function 7: N=6 |  | 2700 |
| **C28** | Function 8: N=6 |  | 2800 |
| **C29** | Function 9: N=3 |  | 2900 |
| **C30** | Function 10: N=3 |  | 3000 |

**Appendix A.2.** Impact assessment of population size.

| **Size pop** | | **10** | | **30** | | | **60** | | | | | **100** | | | | **200** | | |  |
| --- | --- | --- | --- | --- | --- | --- | --- | --- | --- | --- | --- | --- | --- | --- | --- | --- | --- | --- | --- |
| **Metrics** | | Avg | Std | Avg | Std | | Avg | | Std | | | Avg | | Std | | Avg | | Std |  |
| F1 | SMA_FBI | 3.81E+02 | 1.40E+02 | **3.70E+02** | | **6.04E+01** | | 5.74E+02 | | 4.28E+02 | 5.58E+02 | | 2.60E+02 | | 1.27E+03 | | 1.37E+03 | | |
|  | FBI | 3.09E+02 | 4.16E+01 | 3.98E+03 | | 2.26E+03 | | 1.35E+04 | | 3.46E+03 | 1.75E+04 | | 4.05E+03 | | 1.94E+04 | | 3.69E+03 | | |
| F7 | SMA_FBI | 8.41E+02 | 1.62E+01 | **8.21E+02** | | **1.54E+01** | | 8.26E+02 | | 1.61E+01 | 8.27E+02 | | 2.50E+01 | | 8.33E+02 | | 2.99E+01 | | |
|  | FBI | 8.52E+02 | 2.75E+01 | 8.21E+02 | | 1.47E+01 | | 8.27E+02 | | 9.88E+00 | 8.43E+02 | | 8.75E+00 | | 8.87E+02 | | 1.47E+01 | | |
| F14 | SMA_FBI | **1.60E+03** | **7.37E+01** | 1.64E+03 | | 9.33E+01 | | 1.71E+03 | | 7.13E+01 | 1.67E+03 | | 7.96E+01 | | 1.74E+03 | | 2.03E+02 | | |
|  | FBI | 1.65E+03 | 1.72E+02 | 1.80E+03 | | 5.84E+02 | | 2.16E+03 | | 4.78E+02 | 2.64E+03 | | 1.10E+03 | | 4.26E+03 | | 2.18E+03 | | |
| F29 | SMA_FBI | 3.10E+03 | 0.00E+00 | **3.10E+03** | | **0.00E+00** | | 3.10E+03 | | 0.00E+00 | 3.10E+03 | | 0.00E+00 | | 3.10E+03 | | 0.00E+00 | | |
|  | FBI | 3.42E+03 | 1.83E+02 | 3.34E+03 | | 1.19E+02 | | 3.28E+03 | | 1.07E+02 | 3.31E+03 | | 9.81E+01 | | 3.35E+03 | | 1.36E+02 | | |

**Appendix A.3.** Impact of the number of function evaluations.

| **Evaluations** | | **50 000** | | **100 000** | | **150 000** | | **200 000** | | **300 000** | | |
| --- | --- | --- | --- | --- | --- | --- | --- | --- | --- | --- | --- | --- |
| **Metrics** | | Avg | Std | Avg | Std | Avg | Std | Avg | Std | Avg | Std | |
| F3 | SMA_FBI | 4.12E+04 | 7.60E+03 | 1.61E+04 | 7.54E+03 | 5.74E+03 | 4.10E+03 | 1.52E+03 | 9.66E+02 | **4.22E+02** | **1.66E+02** |  |
|  | FBI | 3.84E+04 | 8.33E+03 | 2.76E+04 | 6.82E+03 | 1.79E+04 | 4.99E+03 | 1.12E+04 | 5.03E+03 | 4.26E+03 | 1.34E+03 |  |
| F7 | SMA_FBI | 8.89E+02 | 3.66E+01 | 8.57E+02 | 2.62E+01 | 8.37E+02 | 1.52E+01 | 8.35E+02 | 1.23E+01 | **8.23E+02** | **1.31E+01** |  |
|  | FBI | 9.10E+02 | 2.18E+01 | 8.62E+02 | 1.55E+01 | 8.36E+02 | 1.21E+01 | 8.27E+02 | 1.63E+01 | 8.19E+02 | 1.65E+01 |  |
| F14 | SMA_FBI | 5.20E+03 | 3.34E+03 | 2.56E+03 | 9.65E+02 | 1.94E+03 | 3.88E+02 | 1.73E+03 | 9.70E+01 | **1.64E+03** | **8.68E+01** |  |
|  | FBI | 5.64E+03 | 3.63E+03 | 2.89E+03 | 2.20E+03 | 2.01E+03 | 5.64E+02 | 1.90E+03 | 4.78E+02 | 1.66E+03 | 1.49E+02 |  |
| F29 | SMA_FBI | 3.10E+03 | 0.00E+00 | 3.10E+03 | 0.00E+00 | 3.10E+03 | 0.00E+00 | 3.10E+03 | 0.00E+00 | **3.10E+03** | **0.00E+00** |  |
|  | FBI | 3.34E+03 | 1.72E+02 | 3.34E+03 | 1.24E+02 | 3.31E+03 | 1.33E+02 | 3.30E+03 | 1.28E+02 | 3.27E+03 | 1.30E+02 |  |

**Appendix A.4.** Comparison results with the conventional algorithms.

|  | F1 | |  | F2 | |  | F3 | |
| --- | --- | --- | --- | --- | --- | --- | --- | --- |
|  | Avg | Std |  | Avg | Std |  | Avg | Std |
| SMA_FBI | 1.102E+02 | 1.676E+01 |  | 3.658E+08 | 1.340E+09 |  | 4.595E+02 | 1.748E+02 |
| FBI | **1.032E+02** | **6.702E+00** |  | 4.986E+12 | 2.563E+13 |  | 4.332E+03 | 2.072E+03 |
| SMA | 8.030E+06 | 1.547E+07 |  | 6.944E+17 | 2.298E+18 |  | 3.850E+03 | 1.572E+03 |
| MFO | 1.229E+10 | 7.898E+09 |  | 2.347E+37 | 7.199E+37 |  | 1.010E+05 | 6.613E+04 |
| BA | 4.247E+05 | 2.473E+05 |  | **2.000E+02** | **0.000E+00** |  | **3.001E+02** | **1.556E-01** |
| MVO | 1.331E+04 | 1.681E+04 |  | 4.342E+04 | 9.628E+04 |  | 3.003E+02 | 1.081E-01 |
| GSA | 2.041E+07 | 2.609E+06 |  | 2.196E+19 | 6.603E+19 |  | 7.977E+04 | 1.202E+04 |
| SCA | 1.801E+10 | 4.629E+09 |  | 6.512E+32 | 1.726E+33 |  | 3.588E+04 | 7.086E+03 |
| FA | 1.445E+10 | 1.662E+09 |  | 7.575E+34 | 2.867E+35 |  | 5.560E+04 | 9.977E+03 |
| DE | 3.246E+02 | 6.827E+02 |  | 9.240E+24 | 1.347E+25 |  | 2.181E+04 | 5.632E+03 |
| PSO | 1.432E+08 | 1.332E+07 |  | 4.750E+12 | 1.004E+13 |  | 6.092E+02 | 4.835E+01 |
|  | F4 | |  | F5 | |  | F6 | |
|  | Avg | Std |  | Avg | Std |  | Avg | Std |
| SMA_FBI | **4.278E+02** | **3.156E+01** |  | **5.694E+02** | **1.052E+01** |  | **6.000E+02** | **3.799E-03** |
| FBI | 4.287E+02 | 3.308E+01 |  | 5.696E+02 | 9.536E+00 |  | 6.000E+02 | 2.771E-04 |
| SMA | 5.331E+02 | 3.199E+01 |  | 6.078E+02 | 2.367E+01 |  | 6.165E+02 | 6.298E+00 |
| MFO | 1.948E+03 | 1.774E+03 |  | 7.388E+02 | 5.427E+01 |  | 6.436E+02 | 1.036E+01 |
| BA | 4.290E+02 | 3.775E+01 |  | 7.623E+02 | 5.373E+01 |  | 6.659E+02 | 1.110E+01 |
| MVO | 4.904E+02 | 2.454E+01 |  | 5.908E+02 | 2.423E+01 |  | 6.082E+02 | 5.713E+00 |
| GSA | 4.573E+02 | 5.908E+01 |  | 5.600E+02 | 1.351E+01 |  | 6.016E+02 | 5.754E-01 |
| SCA | 1.536E+03 | 4.322E+02 |  | 7.487E+02 | 2.001E+01 |  | 6.426E+02 | 4.795E+00 |
| FA | 1.549E+03 | 1.647E+02 |  | 7.501E+02 | 1.177E+01 |  | 6.437E+02 | 3.038E+00 |
| DE | 4.841E+02 | 3.063E+01 |  | 6.082E+02 | 8.695E+00 |  | 6.000E+02 | 2.111E-14 |
| PSO | 4.625E+02 | 3.690E+01 |  | 6.985E+02 | 2.009E+01 |  | 6.354E+02 | 1.239E+01 |
|  | F7 | |  | F8 | |  | F9 | |
|  | Avg | Std |  | Avg | Std |  | Avg | Std |
| SMA_FBI | **8.214E+02** | **1.597E+01** |  | 8.676E+02 | 1.032E+01 |  | 1.127E+03 | 2.405E+02 |
| FBI | 8.221E+02 | 1.077E+01 |  | 8.692E+02 | 1.163E+01 |  | 1.108E+03 | 2.542E+02 |
| SMA | 9.582E+02 | 6.497E+01 |  | 9.245E+02 | 2.248E+01 |  | 4.186E+03 | 6.886E+02 |
| MFO | 1.193E+03 | 2.206E+02 |  | 1.013E+03 | 4.248E+01 |  | 7.976E+03 | 2.999E+03 |
| BA | 1.731E+03 | 2.021E+02 |  | 1.134E+03 | 6.696E+01 |  | 1.660E+04 | 4.977E+03 |
| MVO | 8.247E+02 | 2.703E+01 |  | 8.862E+02 | 2.174E+01 |  | 2.732E+03 | 2.394E+03 |
| GSA | 8.526E+02 | 1.000E+01 |  | **8.656E+02** | **9.612E+00** |  | 9.070E+02 | 6.101E-01 |
| SCA | 1.201E+03 | 5.730E+01 |  | 1.072E+03 | 2.574E+01 |  | 7.131E+03 | 1.224E+03 |
| FA | 1.378E+03 | 4.240E+01 |  | 1.054E+03 | 1.515E+01 |  | 5.870E+03 | 6.386E+02 |
| DE | 8.415E+02 | 9.368E+00 |  | 9.118E+02 | 9.463E+00 |  | **9.000E+02** | **1.034E-13** |
| PSO | 9.153E+02 | 1.507E+01 |  | 1.043E+03 | 3.286E+01 |  | 6.881E+03 | 2.156E+03 |
|  | F10 | |  | F11 | |  | F12 | |
|  | Avg | Std |  | Avg | Std |  | Avg | Std |
| SMA_FBI | 3.019E+03 | 3.174E+02 |  | 1.161E+03 | 3.034E+01 |  | 7.569E+03 | 9.656E+03 |
| FBI | 3.036E+03 | 2.960E+02 |  | 1.164E+03 | 2.882E+01 |  | **6.701E+03** | **5.654E+03** |
| SMA | 3.755E+03 | 6.548E+02 |  | 1.254E+03 | 3.801E+01 |  | 3.640E+06 | 2.637E+06 |
| MFO | 5.053E+03 | 8.723E+02 |  | 7.000E+03 | 6.921E+03 |  | 1.053E+09 | 1.566E+09 |
| BA | 5.390E+03 | 7.714E+02 |  | 1.418E+03 | 1.054E+02 |  | 7.853E+06 | 6.656E+06 |
| MVO | 3.911E+03 | 6.177E+02 |  | 1.274E+03 | 7.426E+01 |  | 1.536E+07 | 8.744E+06 |
| GSA | **2.656E+03** | **2.865E+02** |  | 3.377E+03 | 1.398E+03 |  | 3.332E+06 | 1.222E+06 |
| SCA | 7.943E+03 | 3.600E+02 |  | 2.920E+03 | 4.929E+02 |  | 1.482E+09 | 5.691E+08 |
| FA | 7.789E+03 | 2.040E+02 |  | 4.287E+03 | 8.258E+02 |  | 2.557E+09 | 5.210E+08 |
| DE | 5.541E+03 | 3.049E+02 |  | **1.149E+03** | **8.890E+00** |  | 1.149E+04 | 5.639E+03 |
| PSO | 5.612E+03 | 5.279E+02 |  | 1.356E+03 | 5.064E+01 |  | 7.231E+07 | 3.148E+07 |
|  | F13 | |  | F14 | |  | F15 | |
|  | Avg | Std |  | Avg | Std |  | Avg | Std |
| SMA_FBI | 1.475E+03 | 7.336E+01 |  | 1.635E+03 | 8.847E+01 |  | 1.746E+03 | 1.700E+02 |
| FBI | 1.422E+03 | 6.644E+01 |  | 1.676E+03 | 1.893E+02 |  | 1.650E+03 | 8.182E+01 |
| SMA | 1.482E+04 | 4.397E+03 |  | 1.650E+03 | 6.261E+01 |  | 5.322E+03 | 4.231E+03 |
| MFO | 8.888E+07 | 2.019E+08 |  | 5.525E+05 | 1.185E+06 |  | 2.418E+07 | 9.184E+07 |
| BA | 2.033E+05 | 9.617E+04 |  | 1.348E+04 | 7.746E+03 |  | 9.438E+04 | 6.800E+04 |
| MVO | 3.388E+04 | 2.555E+04 |  | 2.750E+03 | 1.495E+03 |  | 2.337E+04 | 1.708E+04 |
| GSA | 1.542E+05 | 5.545E+04 |  | 3.797E+03 | 2.070E+03 |  | 2.748E+04 | 1.254E+04 |
| SCA | 1.173E+08 | 6.060E+07 |  | 2.001E+05 | 1.169E+05 |  | 4.666E+06 | 2.739E+06 |
| FA | 3.791E+08 | 1.314E+08 |  | 2.627E+05 | 1.238E+05 |  | 5.496E+07 | 2.279E+07 |
| DE | **1.353E+03** | **5.008E+01** |  | **1.463E+03** | **7.393E+00** |  | **1.551E+03** | **1.373E+01** |
| PSO | 2.500E+06 | 5.622E+05 |  | 2.581E+04 | 1.397E+04 |  | 3.190E+05 | 9.371E+04 |
|  | F16 | |  | F17 | |  | F18 | |
|  | Avg | Std |  | Avg | Std |  | Avg | Std |
| SMA_FBI | 2.157E+03 | 1.400E+02 |  | 1.935E+03 | 7.324E+01 |  | 1.341E+05 | 8.300E+04 |
| FBI | 2.162E+03 | 1.628E+02 |  | **1.916E+03** | **7.792E+01** |  | **1.007E+05** | **9.122E+04** |
| SMA | 2.491E+03 | 2.714E+02 |  | 2.078E+03 | 1.051E+02 |  | 1.219E+05 | 1.131E+05 |
| MFO | 3.119E+03 | 3.658E+02 |  | 2.401E+03 | 2.216E+02 |  | 5.002E+05 | 1.091E+06 |
| BA | 3.327E+03 | 4.876E+02 |  | 2.946E+03 | 3.541E+02 |  | 7.403E+04 | 3.806E+04 |
| MVO | 2.336E+03 | 2.789E+02 |  | 2.040E+03 | 1.192E+02 |  | 6.024E+04 | 6.263E+04 |
| GSA | 2.981E+03 | 3.396E+02 |  | 2.534E+03 | 2.147E+02 |  | 6.830E+04 | 3.334E+04 |
| SCA | 3.332E+03 | 2.184E+02 |  | 2.530E+03 | 1.796E+02 |  | 1.514E+06 | 9.360E+05 |
| FA | 3.286E+03 | 1.306E+02 |  | 2.501E+03 | 1.325E+02 |  | 1.748E+06 | 8.167E+05 |
| DE | **2.026E+03** | **1.348E+02** |  | 1.960E+03 | 4.028E+01 |  | 3.751E+05 | 2.374E+05 |
| PSO | 2.771E+03 | 2.674E+02 |  | 2.468E+03 | 2.206E+02 |  | 9.041E+04 | 3.738E+04 |
|  | F19 | |  | F20 | |  | F21 | |
|  | Avg | Std |  | Avg | Std |  | Avg | Std |
| SMA_FBI | 3.170E+03 | 1.515E+03 |  | 2.264E+03 | 5.776E+01 |  | **2.250E+03** | **0.000E+00** |
| FBI | **3.054E+03** | **1.467E+03** |  | 2.250E+03 | 8.166E+01 |  | 2.250E+03 | 0.000E+00 |
| SMA | 9.940E+03 | 7.670E+03 |  | 2.348E+03 | 1.323E+02 |  | 2.250E+03 | 0.000E+00 |
| MFO | 8.632E+05 | 2.005E+06 |  | 2.677E+03 | 2.297E+02 |  | 2.251E+03 | 2.891E+00 |
| BA | 2.851E+05 | 1.302E+05 |  | 2.871E+03 | 2.747E+02 |  | 2.250E+03 | 2.665E-04 |
| MVO | 1.350E+04 | 9.696E+03 |  | 2.403E+03 | 1.565E+02 |  | 2.250E+03 | 1.897E-06 |
| GSA | 1.444E+04 | 7.789E+03 |  | 3.014E+03 | 1.884E+02 |  | 2.250E+03 | 6.301E-04 |
| SCA | 2.155E+07 | 1.552E+07 |  | 2.703E+03 | 9.937E+01 |  | 2.250E+03 | 1.194E-13 |
| FA | 3.163E+07 | 1.384E+07 |  | 2.610E+03 | 9.047E+01 |  | 2.257E+03 | 7.071E-01 |
| DE | 5.438E+03 | 2.428E+03 |  | **2.199E+03** | **3.337E+01** |  | 2.250E+03 | 4.306E-13 |
| PSO | 3.092E+05 | 2.320E+05 |  | 2.622E+03 | 1.600E+02 |  | 2.250E+03 | 5.345E-03 |
|  | F22 | |  | F23 | |  | F24 | |
|  | Avg | Std |  | Avg | Std |  | Avg | Std |
| SMA_FBI | **2.350E+03** | **0.000E+00** |  | **2.500E+03** | **0.000E+00** |  | **2.600E+03** | **0.000E+00** |
| FBI | 2.350E+03 | 0.000E+00 |  | 2.887E+03 | 2.754E+01 |  | 2.600E+03 | 0.000E+00 |
| SMA | 2.350E+03 | 0.000E+00 |  | 2.500E+03 | 0.000E+00 |  | 2.600E+03 | 0.000E+00 |
| MFO | 2.351E+03 | 2.886E+00 |  | 2.965E+03 | 2.765E+01 |  | 3.510E+03 | 4.169E+01 |
| BA | 2.350E+03 | 2.766E-04 |  | 3.465E+03 | 1.660E+02 |  | 2.877E+03 | 5.447E+02 |
| MVO | 2.350E+03 | 5.055E-07 |  | 2.867E+03 | 2.430E+01 |  | 3.419E+03 | 2.241E+01 |
| GSA | 2.350E+03 | 1.104E-03 |  | 3.240E+03 | 1.581E+02 |  | 2.630E+03 | 9.510E-01 |
| SCA | 2.350E+03 | 8.444E-14 |  | 3.279E+03 | 4.562E+01 |  | 3.870E+03 | 6.969E+01 |
| FA | 2.357E+03 | 5.542E-01 |  | 3.104E+03 | 1.314E+01 |  | 3.685E+03 | 2.047E+01 |
| DE | 2.350E+03 | 4.137E-13 |  | 2.872E+03 | 8.578E+00 |  | 3.397E+03 | 8.266E+00 |
| PSO | 2.350E+03 | 6.198E-03 |  | 4.775E+03 | 5.754E+02 |  | 2.667E+03 | 3.767E+00 |
|  | F25 | |  | F26 | |  | F27 | |
|  | Avg | Std |  | Avg | Std |  | Avg | Std |
| SMA_FBI | **2.700E+03** | **0.000E+00** |  | **2.800E+03** | **0.000E+00** |  | **2.900E+03** | **0.000E+00** |
| FBI | 2.700E+03 | 0.000E+00 |  | 2.800E+03 | 0.000E+00 |  | 3.742E+03 | 7.892E+01 |
| SMA | 2.700E+03 | 0.000E+00 |  | 2.800E+03 | 0.000E+00 |  | 2.900E+03 | 0.000E+00 |
| MFO | 3.516E+03 | 6.720E+02 |  | 6.625E+03 | 6.382E+02 |  | 3.593E+03 | 7.760E+01 |
| BA | 3.015E+03 | 5.268E+01 |  | 5.390E+03 | 3.790E+03 |  | 3.916E+03 | 2.134E+02 |
| MVO | 2.926E+03 | 3.048E+01 |  | 5.181E+03 | 2.396E+02 |  | 3.617E+03 | 1.236E+02 |
| GSA | 2.941E+03 | 1.876E+01 |  | 3.039E+03 | 1.522E+01 |  | 4.364E+03 | 3.015E+02 |
| SCA | 3.595E+03 | 2.327E+02 |  | 7.956E+03 | 3.129E+02 |  | 4.062E+03 | 9.768E+01 |
| FA | 4.129E+03 | 1.222E+02 |  | 7.244E+03 | 1.376E+02 |  | 3.886E+03 | 5.388E+01 |
| DE | 2.912E+03 | 4.703E+00 |  | 5.385E+03 | 7.951E+01 |  | 3.434E+03 | 1.831E+01 |
| PSO | 2.990E+03 | 1.081E+02 |  | 3.410E+03 | 3.637E+01 |  | 4.762E+03 | 7.189E+02 |
|  | F28 | |  | F29 | |  | F30 | |
|  | Avg | Std |  | Avg | Std |  | Avg | Std |
| SMA_FBI | **3.000E+03** | **0.000E+00** |  | **3.100E+03** | **0.000E+00** |  | **3.200E+03** | **0.000E+00** |
| FBI | 3.000E+03 | 0.000E+00 |  | 3.311E+03 | 1.041E+02 |  | 1.057E+04 | 1.202E+04 |
| SMA | 3.000E+03 | 0.000E+00 |  | 3.100E+03 | 0.000E+00 |  | 1.518E+04 | 1.516E+04 |
| MFO | 5.005E+03 | 6.006E+02 |  | 4.145E+03 | 2.996E+02 |  | 1.444E+06 | 1.720E+06 |
| BA | 3.385E+03 | 5.065E+02 |  | 4.597E+03 | 4.536E+02 |  | 1.354E+06 | 9.290E+05 |
| MVO | 3.761E+03 | 8.842E+02 |  | 3.677E+03 | 1.621E+02 |  | 1.330E+06 | 1.387E+06 |
| GSA | 3.349E+03 | 6.666E+01 |  | 3.494E+03 | 1.906E+02 |  | 3.111E+05 | 1.219E+05 |
| SCA | 5.577E+03 | 7.322E+02 |  | 4.225E+03 | 2.946E+02 |  | 8.570E+06 | 1.685E+07 |
| FA | 4.061E+03 | 7.561E+01 |  | 4.546E+03 | 1.704E+02 |  | 1.063E+08 | 2.863E+07 |
| DE | 3.786E+03 | 6.904E+02 |  | 3.483E+03 | 7.456E+01 |  | 6.114E+04 | 2.491E+04 |
| PSO | 3.320E+03 | 1.291E+02 |  | 4.032E+03 | 2.290E+02 |  | 2.824E+06 | 1.269E+06 |
|  | Statistical Comparison | |  |  |  |  |  |  |
|  | SMA_FBI | FBI |  | SMA | MFO |  | BA | MVO |
| +/-/= | ~ | 6/5/19 |  | 19/0/11 | 29/0/1 |  | 26/3/1 | 26/3/1 |
| ARV | 2.455 | 2.759 |  | 4.036 | 7.955 |  | 7.389 | 4.904 |
| Rank | 1 | 2 |  | 3 | 9 |  | 7 | 5 |
|  | GSA | SCA |  | FA | DE |  | PSO |  |
| +/-/= | 25/4/1 | 28/0/2 |  | 30/0/0 | 18/7/5 |  | 29/0/1 |  |
| ARV | 6.071 | 9.08 |  | 9.698 | 4.169 |  | 7.484 |  |
| Rank | 6 | 10 |  | 11 | 4 |  | 8 |  |

**Appendix A.5.** The p-value of Wilcoxon test between the SMA_FBI and conventional algorithms.

| Function | FBI | SMA | MFO | BA | MVO | GSA | SCA | FA | DE | PSO |
| --- | --- | --- | --- | --- | --- | --- | --- | --- | --- | --- |
| F1 | 4.28E-02 | 1.73E-06 | 1.73E-06 | 1.73E-06 | 1.73E-06 | 1.73E-06 | 1.73E-06 | 1.73E-06 | 7.52E-02 | 1.73E-06 |
| F2 | 2.60E-06 | 1.73E-06 | 1.73E-06 | 1.73E-06 | 5.79E-05 | 1.73E-06 | 1.73E-06 | 1.73E-06 | 1.73E-06 | 1.73E-06 |
| F3 | 1.73E-06 | 1.73E-06 | 1.73E-06 | 1.73E-06 | 1.73E-06 | 1.73E-06 | 1.73E-06 | 1.73E-06 | 1.73E-06 | 8.92E-05 |
| F4 | 7.66E-01 | 1.73E-06 | 1.73E-06 | 8.77E-01 | 2.88E-06 | 4.72E-02 | 1.73E-06 | 1.73E-06 | 4.86E-05 | 6.84E-03 |
| F5 | 9.43E-01 | 2.35E-06 | 1.73E-06 | 1.73E-06 | 8.92E-05 | 5.67E-03 | 1.73E-06 | 1.73E-06 | 1.73E-06 | 1.73E-06 |
| F6 | 1.92E-06 | 1.73E-06 | 1.73E-06 | 1.73E-06 | 1.73E-06 | 1.73E-06 | 1.73E-06 | 1.73E-06 | 1.73E-06 | 1.73E-06 |
| F7 | 8.13E-01 | 1.73E-06 | 1.73E-06 | 1.73E-06 | 7.66E-01 | 4.29E-06 | 1.73E-06 | 1.73E-06 | 4.45E-05 | 1.73E-06 |
| F8 | 6.88E-01 | 1.73E-06 | 1.73E-06 | 1.73E-06 | 3.32E-04 | 2.90E-01 | 1.73E-06 | 1.73E-06 | 1.73E-06 | 1.73E-06 |
| F9 | 3.71E-01 | 1.73E-06 | 1.73E-06 | 1.73E-06 | 2.96E-03 | 1.73E-06 | 1.73E-06 | 1.73E-06 | 1.73E-06 | 1.73E-06 |
| F10 | 9.10E-01 | 2.84E-05 | 1.73E-06 | 1.73E-06 | 1.24E-05 | 6.64E-04 | 1.73E-06 | 1.73E-06 | 1.73E-06 | 1.73E-06 |
| F11 | 7.50E-01 | 5.22E-06 | 1.73E-06 | 1.73E-06 | 1.73E-06 | 1.73E-06 | 1.73E-06 | 1.73E-06 | 1.47E-01 | 1.73E-06 |
| F12 | 9.59E-01 | 1.73E-06 | 1.73E-06 | 1.73E-06 | 1.73E-06 | 1.73E-06 | 1.73E-06 | 1.73E-06 | 7.16E-04 | 1.73E-06 |
| F13 | 3.00E-02 | 1.73E-06 | 1.73E-06 | 1.73E-06 | 1.73E-06 | 1.73E-06 | 1.73E-06 | 1.73E-06 | 1.97E-05 | 1.73E-06 |
| F14 | 4.65E-01 | 6.00E-01 | 1.73E-06 | 1.73E-06 | 3.18E-06 | 1.73E-06 | 1.73E-06 | 1.73E-06 | 1.73E-06 | 1.73E-06 |
| F15 | 5.32E-03 | 1.73E-06 | 1.73E-06 | 1.73E-06 | 1.73E-06 | 1.73E-06 | 1.73E-06 | 1.73E-06 | 1.73E-06 | 1.73E-06 |
| F16 | 7.04E-01 | 1.97E-05 | 1.73E-06 | 1.73E-06 | 4.68E-03 | 1.73E-06 | 1.73E-06 | 1.73E-06 | 2.26E-03 | 1.73E-06 |
| F17 | 5.86E-01 | 1.06E-04 | 1.73E-06 | 1.73E-06 | 8.94E-04 | 1.73E-06 | 1.73E-06 | 1.73E-06 | 1.71E-01 | 1.73E-06 |
| F18 | 2.30E-02 | 4.28E-01 | 2.21E-01 | 6.64E-04 | 3.41E-05 | 2.61E-04 | 1.73E-06 | 1.73E-06 | 7.51E-05 | 7.19E-02 |
| F19 | 4.28E-01 | 1.06E-04 | 5.22E-06 | 1.73E-06 | 3.11E-05 | 2.88E-06 | 1.73E-06 | 1.73E-06 | 5.29E-04 | 1.73E-06 |
| F20 | 4.91E-01 | 4.68E-03 | 1.73E-06 | 1.73E-06 | 1.48E-04 | 1.73E-06 | 1.73E-06 | 1.73E-06 | 6.32E-05 | 1.73E-06 |
| F21 | 1.00E+00 | 1.00E+00 | 3.13E-02 | 1.73E-06 | 1.73E-06 | 1.73E-06 | 1.00E+00 | 1.73E-06 | 1.00E+00 | 1.73E-06 |
| F22 | 1.00E+00 | 1.00E+00 | 3.13E-02 | 1.73E-06 | 1.73E-06 | 1.73E-06 | 1.00E+00 | 1.73E-06 | 1.00E+00 | 1.73E-06 |
| F23 | 1.73E-06 | 1.00E+00 | 1.73E-06 | 1.73E-06 | 1.73E-06 | 1.73E-06 | 1.73E-06 | 1.73E-06 | 1.73E-06 | 1.73E-06 |
| F24 | 1.00E+00 | 1.00E+00 | 1.73E-06 | 1.73E-06 | 1.73E-06 | 1.73E-06 | 1.73E-06 | 1.73E-06 | 1.73E-06 | 1.73E-06 |
| F25 | 1.00E+00 | 1.00E+00 | 1.73E-06 | 1.73E-06 | 1.73E-06 | 1.73E-06 | 2.56E-06 | 1.73E-06 | 1.73E-06 | 1.73E-06 |
| F26 | 1.00E+00 | 1.00E+00 | 1.73E-06 | 1.73E-06 | 1.73E-06 | 1.73E-06 | 1.73E-06 | 1.73E-06 | 1.73E-06 | 1.73E-06 |
| F27 | 1.73E-06 | 1.00E+00 | 1.73E-06 | 1.73E-06 | 1.73E-06 | 1.73E-06 | 1.73E-06 | 1.73E-06 | 1.73E-06 | 1.73E-06 |
| F28 | 1.00E+00 | 1.00E+00 | 1.64E-06 | 1.73E-06 | 1.73E-06 | 1.73E-06 | 1.73E-06 | 1.73E-06 | 1.72E-06 | 1.73E-06 |
| F29 | 1.23E-05 | 1.00E+00 | 1.73E-06 | 1.73E-06 | 1.73E-06 | 1.73E-06 | 1.73E-06 | 1.73E-06 | 1.73E-06 | 1.73E-06 |
| F30 | 2.93E-04 | 4.38E-04 | 1.73E-06 | 1.73E-06 | 1.73E-06 | 1.73E-06 | 1.73E-06 | 1.73E-06 | 1.73E-06 | 1.73E-06 |

**Appendix A.6.** Comparison results with state-of-the-art algorithms.

|  | F1 | |  | F2 | |  | F3 | |
| --- | --- | --- | --- | --- | --- | --- | --- | --- |
|  | Avg | Std |  | Avg | Std |  | Avg | Std |
| SMA_FBI | 1.164E+02 | 2.537E+01 |  | 2.401E+08 | 9.617E+08 |  | 3.785E+02 | 9.639E+01 |
| EPSDE | 1.000E+02 | 6.841E-14 |  | 9.240E+19 | 5.015E+20 |  | **3.000E+02** | **1.439E-05** |
| ALCPSO | 1.436E+03 | 1.837E+03 |  | 3.058E+21 | 1.102E+22 |  | 2.599E+04 | 4.066E+03 |
| BMWOA | 1.728E+08 | 8.024E+07 |  | 1.252E+28 | 3.997E+28 |  | 5.471E+04 | 1.106E+04 |
| CLPSO | 1.057E+02 | 1.056E+01 |  | 3.431E+13 | 9.840E+13 |  | 8.784E+03 | 2.099E+03 |
| IGWO | 3.320E+06 | 1.804E+06 |  | 7.784E+14 | 1.784E+15 |  | 8.985E+02 | 3.889E+02 |
| CESCA | 9.797E+10 | 7.067E+09 |  | 2.547E+43 | 1.101E+44 |  | 1.105E+05 | 2.162E+04 |
| RDWOA | 9.657E+06 | 1.347E+07 |  | 6.996E+17 | 2.070E+18 |  | 8.278E+03 | 3.977E+03 |
| LSHADE | **1.000E+02** | **5.889E-14** |  | 1.835E+14 | 1.002E+15 |  | 2.006E+04 | 3.455E+04 |
| CBA | 1.351E+04 | 4.058E+04 |  | **7.280E+03** | **7.862E+03** |  | 3.147E+02 | 8.320E+00 |
| DECLS | 7.474E+02 | 2.238E+03 |  | 1.198E+27 | 2.490E+27 |  | 5.361E+04 | 1.409E+04 |
|  | F4 | |  | F5 | |  | F6 | |
|  | Avg | Std |  | Avg | Std |  | Avg | Std |
| SMA_FBI | 4.299E+02 | 3.238E+01 |  | 5.702E+02 | 1.023E+01 |  | **6.000E+02** | **2.096E-03** |
| EPSDE | **4.134E+02** | **1.207E+01** |  | 5.435E+02 | 7.385E+00 |  | 6.000E+02 | 2.499E-08 |
| ALCPSO | 5.243E+02 | 5.189E+01 |  | 6.066E+02 | 2.761E+01 |  | 6.075E+02 | 7.437E+00 |
| BMWOA | 6.901E+02 | 6.078E+01 |  | 7.072E+02 | 2.793E+01 |  | 6.575E+02 | 6.124E+00 |
| CLPSO | 4.648E+02 | 2.256E+01 |  | 5.534E+02 | 9.767E+00 |  | 6.000E+02 | 7.313E-14 |
| IGWO | 5.228E+02 | 3.256E+01 |  | 5.892E+02 | 1.988E+01 |  | 6.218E+02 | 5.888E+00 |
| CESCA | 1.207E+04 | 1.888E+03 |  | 8.990E+02 | 1.547E+01 |  | 6.880E+02 | 4.518E+00 |
| RDWOA | 5.446E+02 | 4.311E+01 |  | 6.587E+02 | 3.018E+01 |  | 6.102E+02 | 3.683E+00 |
| LSHADE | 4.045E+02 | 1.625E+01 |  | **5.347E+02** | **8.591E+00** |  | 6.002E+02 | 2.327E-01 |
| CBA | 5.100E+02 | 4.332E+01 |  | 7.431E+02 | 5.743E+01 |  | 6.660E+02 | 1.100E+01 |
| DECLS | 5.164E+02 | 2.148E+01 |  | 6.261E+02 | 8.945E+00 |  | 6.000E+02 | 4.222E-14 |
|  | F7 | |  | F8 | |  | F9 | |
|  | Avg | Std |  | Avg | Std |  | Avg | Std |
| SMA_FBI | 8.161E+02 | 2.069E+01 |  | 8.712E+02 | 9.334E+00 |  | 1.051E+03 | 1.439E+02 |
| EPSDE | **7.707E+02** | **8.989E+00** |  | 8.389E+02 | 7.038E+00 |  | 9.007E+02 | 1.880E+00 |
| ALCPSO | 8.426E+02 | 3.132E+01 |  | 9.050E+02 | 2.571E+01 |  | 2.097E+03 | 1.099E+03 |
| BMWOA | 1.239E+03 | 9.544E+01 |  | 1.062E+03 | 5.328E+01 |  | 8.994E+03 | 1.682E+03 |
| CLPSO | 7.841E+02 | 8.087E+00 |  | 8.487E+02 | 8.851E+00 |  | 9.201E+02 | 2.550E+01 |
| IGWO | 9.195E+02 | 4.645E+01 |  | 8.993E+02 | 1.689E+01 |  | 3.279E+03 | 8.345E+02 |
| CESCA | 1.764E+03 | 4.960E+01 |  | 1.266E+03 | 2.549E+01 |  | 1.750E+04 | 1.575E+03 |
| RDWOA | 9.938E+02 | 6.468E+01 |  | 9.979E+02 | 4.749E+01 |  | 5.236E+03 | 1.499E+03 |
| LSHADE | 7.842E+02 | 1.842E+01 |  | **8.310E+02** | **6.870E+00** |  | 1.011E+03 | 1.215E+02 |
| CBA | 2.106E+03 | 3.390E+02 |  | 1.120E+03 | 6.439E+01 |  | 1.000E+04 | 3.137E+03 |
| DECLS | 8.595E+02 | 9.661E+00 |  | 9.229E+02 | 9.810E+00 |  | **9.000E+02** | **2.986E-14** |
|  | F10 | |  | F11 | |  | F12 | |
|  | Avg | Std |  | Avg | Std |  | Avg | Std |
| SMA_FBI | 3.061E+03 | 3.072E+02 |  | 1.160E+03 | 3.310E+01 |  | 7.492E+03 | 5.975E+03 |
| EPSDE | 4.230E+03 | 5.726E+02 |  | 1.194E+03 | 1.203E+02 |  | 4.926E+03 | 5.037E+03 |
| ALCPSO | 4.094E+03 | 7.267E+02 |  | 1.244E+03 | 5.915E+01 |  | 5.297E+03 | 3.223E+03 |
| BMWOA | 6.658E+03 | 8.985E+02 |  | 3.322E+03 | 7.122E+02 |  | 1.614E+08 | 1.043E+08 |
| CLPSO | 2.948E+03 | 2.794E+02 |  | **1.145E+03** | **1.496E+01** |  | 5.958E+05 | 1.186E+06 |
| IGWO | 4.228E+03 | 6.729E+02 |  | 1.317E+03 | 4.389E+01 |  | 8.440E+07 | 2.640E+07 |
| CESCA | 9.276E+03 | 2.739E+02 |  | 2.520E+04 | 7.669E+03 |  | 2.760E+10 | 3.420E+09 |
| RDWOA | 4.626E+03 | 6.279E+02 |  | 1.316E+03 | 6.303E+01 |  | 2.538E+07 | 2.757E+07 |
| LSHADE | **2.804E+03** | **6.246E+02** |  | 1.306E+03 | 9.530E+01 |  | **2.761E+03** | **3.676E+02** |
| CBA | 5.436E+03 | 5.931E+02 |  | 1.506E+03 | 1.216E+02 |  | 4.133E+07 | 2.054E+07 |
| DECLS | 5.947E+03 | 2.989E+02 |  | 1.177E+03 | 1.216E+01 |  | 1.456E+05 | 3.345E+05 |
|  | F13 | |  | F14 | |  | F15 | |
|  | Avg | Std |  | Avg | Std |  | Avg | Std |
| SMA_FBI | 1.485E+03 | 8.718E+01 |  | 1.623E+03 | 8.220E+01 |  | 1.750E+03 | 2.573E+02 |
| EPSDE | **1.342E+03** | **2.446E+01** |  | 1.518E+03 | 1.251E+02 |  | 1.736E+03 | 2.125E+02 |
| ALCPSO | 2.205E+03 | 1.127E+03 |  | 1.534E+03 | 9.315E+01 |  | 1.632E+03 | 4.535E+01 |
| BMWOA | 2.351E+05 | 1.882E+05 |  | 6.613E+05 | 5.479E+05 |  | 3.685E+04 | 2.984E+04 |
| CLPSO | 1.410E+03 | 5.289E+01 |  | 7.164E+03 | 8.933E+03 |  | 1.590E+03 | 3.880E+01 |
| IGWO | 8.691E+04 | 6.788E+04 |  | 3.406E+04 | 3.258E+04 |  | 2.720E+04 | 1.714E+04 |
| CESCA | 4.332E+09 | 1.495E+09 |  | 6.501E+06 | 2.785E+06 |  | 4.203E+08 | 1.614E+08 |
| RDWOA | 6.051E+03 | 5.154E+03 |  | 7.510E+03 | 2.991E+04 |  | 4.730E+03 | 3.940E+03 |
| LSHADE | 1.610E+03 | 4.046E+02 |  | 1.606E+03 | 9.396E+01 |  | 1.619E+03 | 5.588E+01 |
| CBA | 1.217E+05 | 1.361E+05 |  | 3.623E+04 | 3.388E+04 |  | 8.775E+04 | 8.948E+04 |
| DECLS | 1.381E+03 | 8.945E+01 |  | **1.502E+03** | **1.582E+01** |  | **1.577E+03** | **1.803E+01** |
|  | F16 | |  | F17 | |  | F18 | |
|  | Avg | Std |  | Avg | Std |  | Avg | Std |
| SMA_FBI | 2.162E+03 | 1.558E+02 |  | 1.931E+03 | 7.536E+01 |  | 8.526E+04 | 5.164E+04 |
| EPSDE | 2.111E+03 | 1.693E+02 |  | 1.986E+03 | 6.242E+01 |  | 3.938E+03 | 2.589E+03 |
| ALCPSO | 2.467E+03 | 3.039E+02 |  | 2.091E+03 | 1.298E+02 |  | 4.154E+05 | 6.720E+05 |
| BMWOA | 3.164E+03 | 4.557E+02 |  | 2.544E+03 | 2.581E+02 |  | 2.285E+06 | 1.508E+06 |
| CLPSO | **2.019E+03** | **1.255E+02** |  | 1.918E+03 | 4.255E+01 |  | 1.126E+05 | 9.823E+04 |
| IGWO | 2.475E+03 | 2.044E+02 |  | 2.079E+03 | 1.571E+02 |  | 2.481E+05 | 2.846E+05 |
| CESCA | 5.345E+03 | 3.996E+02 |  | 4.089E+03 | 4.885E+02 |  | 1.887E+07 | 5.564E+06 |
| RDWOA | 2.691E+03 | 3.166E+02 |  | 2.229E+03 | 1.558E+02 |  | 7.904E+05 | 9.215E+05 |
| LSHADE | 2.058E+03 | 1.569E+02 |  | **1.909E+03** | **9.004E+01** |  | **2.010E+03** | **7.702E+01** |
| CBA | 3.516E+03 | 4.639E+02 |  | 2.855E+03 | 4.036E+02 |  | 1.360E+05 | 9.767E+04 |
| DECLS | 2.103E+03 | 1.373E+02 |  | 1.993E+03 | 5.381E+01 |  | 5.659E+05 | 3.301E+05 |
|  | F19 | |  | F20 | |  | F21 | |
|  | Avg | Std |  | Avg | Std |  | Avg | Std |
| SMA_FBI | 3.663E+03 | 1.908E+03 |  | 2.278E+03 | 7.354E+01 |  | 2.170E+03 | 2.132E+01 |
| EPSDE | 2.105E+03 | 6.369E+02 |  | **2.195E+03** | **5.054E+01** |  | **2.108E+03** | **4.962E+00** |
| ALCPSO | 8.557E+03 | 5.914E+03 |  | 2.363E+03 | 1.661E+02 |  | 2.222E+03 | 3.891E+01 |
| BMWOA | 1.004E+05 | 1.296E+05 |  | 2.747E+03 | 1.947E+02 |  | 2.337E+03 | 6.356E+01 |
| CLPSO | **1.972E+03** | **5.632E+01** |  | 2.216E+03 | 5.794E+01 |  | 2.179E+03 | 7.246E+00 |
| IGWO | 1.325E+05 | 1.556E+05 |  | 2.380E+03 | 1.055E+02 |  | 2.234E+03 | 2.292E+01 |
| CESCA | 1.176E+09 | 3.961E+08 |  | 3.162E+03 | 9.095E+01 |  | 1.687E+04 | 1.961E+03 |
| RDWOA | 7.818E+03 | 7.074E+03 |  | 2.422E+03 | 1.315E+02 |  | 2.247E+03 | 2.268E+01 |
| LSHADE | 2.046E+03 | 8.883E+01 |  | 2.199E+03 | 9.305E+01 |  | 2.111E+03 | 2.862E+01 |
| CBA | 4.290E+05 | 3.182E+05 |  | 2.958E+03 | 2.327E+02 |  | 2.225E+03 | 3.090E+01 |
| DECLS | 1.120E+04 | 7.799E+03 |  | 2.270E+03 | 5.023E+01 |  | 2.203E+03 | 2.075E+01 |
|  | F22 | |  | F23 | |  | F24 | |
|  | Avg | Std |  | Avg | Std |  | Avg | Std |
| SMA_FBI | 2.278E+03 | 9.417E+00 |  | **2.500E+03** | **0.000E+00** |  | 2.600E+03 | 0.000E+00 |
| EPSDE | 2.245E+03 | 8.083E+00 |  | 2.697E+03 | 7.994E+00 |  | 2.857E+03 | 9.981E+00 |
| ALCPSO | 2.310E+03 | 3.374E+01 |  | 3.071E+03 | 1.633E+02 |  | 3.269E+03 | 3.807E+02 |
| BMWOA | 2.452E+03 | 2.922E+01 |  | 2.500E+03 | 4.976E-02 |  | 2.600E+03 | 1.029E-01 |
| CLPSO | 2.253E+03 | 7.423E+00 |  | 2.844E+03 | 9.734E+00 |  | 2.609E+03 | 2.547E+01 |
| IGWO | 2.308E+03 | 2.161E+01 |  | 2.912E+03 | 3.801E+01 |  | 3.412E+03 | 1.488E+02 |
| CESCA | 2.630E+03 | 2.444E+01 |  | 3.514E+03 | 7.036E+01 |  | 2.897E+03 | 1.069E+02 |
| RDWOA | 2.393E+03 | 3.335E+01 |  | 2.547E+03 | 1.425E+02 |  | **2.597E+03** | **1.539E+01** |
| LSHADE | **2.232E+03** | **7.261E+00** |  | 2.834E+03 | 1.292E+01 |  | 3.228E+03 | 3.162E+02 |
| CBA | 2.496E+03 | 5.368E+01 |  | 3.503E+03 | 2.423E+02 |  | 2.806E+03 | 5.141E+02 |
| DECLS | 2.327E+03 | 1.125E+01 |  | 2.539E+03 | 1.198E+02 |  | 2.600E+03 | 2.460E-04 |
|  | F25 | |  | F26 | |  | F27 | |
|  | Avg | Std |  | Avg | Std |  | Avg | Std |
| SMA_FBI | **2.700E+03** | **0.000E+00** |  | **2.800E+03** | **0.000E+00** |  | **2.900E+03** | **0.000E+00** |
| EPSDE | 2.953E+03 | 4.041E+01 |  | 3.907E+03 | 1.101E+02 |  | 3.200E+03 | 1.292E-04 |
| ALCPSO | 2.982E+03 | 4.800E+01 |  | 5.297E+03 | 1.312E+03 |  | 3.896E+03 | 1.892E+02 |
| BMWOA | 2.701E+03 | 1.366E+00 |  | 2.801E+03 | 1.113E+00 |  | 2.901E+03 | 6.768E-01 |
| CLPSO | 2.911E+03 | 1.592E+01 |  | 3.806E+03 | 8.583E+02 |  | 3.504E+03 | 3.119E+01 |
| IGWO | 2.983E+03 | 8.601E+01 |  | 5.472E+03 | 7.226E+02 |  | 3.595E+03 | 8.025E+01 |
| CESCA | 4.206E+03 | 5.081E+02 |  | 4.678E+03 | 6.302E+02 |  | 3.819E+03 | 5.007E+01 |
| RDWOA | 2.709E+03 | 4.816E+01 |  | 2.809E+03 | 4.856E+01 |  | 2.988E+03 | 2.710E+02 |
| LSHADE | 2.956E+03 | 4.883E+01 |  | 4.634E+03 | 7.187E+02 |  | 3.575E+03 | 6.772E+01 |
| CBA | 3.035E+03 | 5.888E+01 |  | 5.690E+03 | 3.997E+03 |  | 3.911E+03 | 2.496E+02 |
| DECLS | 2.700E+03 | 3.340E-03 |  | 2.800E+03 | 3.611E-03 |  | 3.049E+03 | 2.508E+02 |
|  | F28 | |  | F29 | |  | F30 | |
|  | Avg | Std |  | Avg | Std |  | Avg | Std |
| SMA_FBI | **3.000E+03** | **0.000E+00** |  | **3.100E+03** | **0.000E+00** |  | **3.200E+03** | **0.000E+00** |
| EPSDE | 3.300E+03 | 8.769E-05 |  | 3.321E+03 | 9.978E+01 |  | 3.256E+03 | 8.930E+01 |
| ALCPSO | 3.341E+03 | 3.619E+02 |  | 3.749E+03 | 2.137E+02 |  | 9.612E+04 | 1.387E+05 |
| BMWOA | 3.002E+03 | 1.765E+00 |  | 3.102E+03 | 1.872E+00 |  | 2.570E+05 | 2.028E+05 |
| CLPSO | 3.284E+03 | 1.503E+01 |  | 3.343E+03 | 5.108E+01 |  | 1.407E+04 | 6.708E+03 |
| IGWO | 4.121E+03 | 9.901E+02 |  | 3.634E+03 | 1.696E+02 |  | 6.484E+05 | 9.503E+05 |
| CESCA | 5.442E+03 | 7.770E+02 |  | 4.256E+03 | 1.969E+02 |  | 8.437E+07 | 3.997E+07 |
| RDWOA | 3.030E+03 | 9.348E+01 |  | 3.147E+03 | 1.862E+02 |  | 8.353E+04 | 5.597E+04 |
| LSHADE | 3.315E+03 | 3.544E+02 |  | 3.367E+03 | 9.820E+01 |  | 5.248E+03 | 4.075E+03 |
| CBA | 3.715E+03 | 1.010E+03 |  | 4.814E+03 | 3.768E+02 |  | 2.284E+06 | 1.375E+06 |
| DECLS | 3.000E+03 | 3.927E-03 |  | 3.100E+03 | 2.753E-01 |  | 9.094E+03 | 2.772E+04 |
|  | Statistical Comparison | |  |  | |  |  | |
|  | SMA_FBI | EPSDE |  | ALCPSO | BMWOA |  | CLPSO | IGWO |
| +/-/= | ~ | 10/15/5 |  | 27/2/1 | 30/0/0 |  | 13/12/5 | 30/0/0 |
| ARV | 3.229 | 3.359 |  | 6.422 | 8.108 |  | 4.243 | 7.307 |
| Rank | 1 | 2 |  | 7 | 9 |  | 4 | 8 |
|  | CESCA | RDWOA |  | LSHADE | CBA |  | DECLS |  |
| +/-/= | 30/0/0 | 22/0/8 |  | 11/14/5 | 28/2/0 |  | 22/5/3 |  |
| ARV | 10.664 | 5.947 |  | 3.790 | 8.236 |  | 4.694 |  |
| Rank | 11 | 6 |  | 3 | 10 |  | 5 |  |

**Appendix A.7.** The p-value of Wilcoxon test between the SMA_FBI and state-of-the-art algorithms.

| Function | EPSDE | ALCPSO | BMWOA | CLPSO | IGWO | CESCA | RDWOA | LSHADE | CBA | DECLS |
| --- | --- | --- | --- | --- | --- | --- | --- | --- | --- | --- |
| F1 | 1.73E-06 | 2.35E-06 | 1.73E-06 | 1.16E-01 | 1.73E-06 | 1.73E-06 | 1.73E-06 | 1.73E-06 | 1.73E-06 | 1.99E-01 |
| F2 | 2.37E-01 | 1.73E-06 | 1.73E-06 | 1.73E-06 | 1.73E-06 | 1.73E-06 | 1.73E-06 | 2.16E-05 | 6.34E-06 | 1.73E-06 |
| F3 | 1.73E-06 | 1.73E-06 | 1.73E-06 | 1.73E-06 | 2.35E-06 | 1.73E-06 | 1.73E-06 | 6.73E-01 | 6.98E-06 | 1.73E-06 |
| F4 | 7.87E-02 | 1.73E-06 | 1.73E-06 | 4.53E-04 | 3.52E-06 | 1.73E-06 | 1.92E-06 | 3.72E-05 | 1.73E-06 | 2.35E-06 |
| F5 | 1.73E-06 | 3.88E-06 | 1.73E-06 | 6.32E-05 | 2.41E-04 | 1.73E-06 | 1.73E-06 | 1.73E-06 | 1.73E-06 | 1.73E-06 |
| F6 | 1.73E-06 | 1.73E-06 | 1.73E-06 | 1.73E-06 | 1.73E-06 | 1.73E-06 | 1.73E-06 | 4.29E-06 | 1.73E-06 | 1.73E-06 |
| F7 | 2.35E-06 | 8.31E-04 | 1.73E-06 | 1.13E-05 | 1.73E-06 | 1.73E-06 | 1.73E-06 | 1.97E-05 | 1.73E-06 | 1.73E-06 |
| F8 | 1.73E-06 | 3.52E-06 | 1.73E-06 | 1.73E-06 | 1.73E-06 | 1.73E-06 | 1.73E-06 | 1.73E-06 | 1.73E-06 | 1.73E-06 |
| F9 | 1.73E-06 | 6.34E-06 | 1.73E-06 | 6.34E-06 | 1.73E-06 | 1.73E-06 | 1.73E-06 | 9.37E-02 | 1.73E-06 | 1.73E-06 |
| F10 | 1.73E-06 | 7.69E-06 | 1.73E-06 | 8.97E-02 | 6.34E-06 | 1.73E-06 | 1.73E-06 | 2.43E-02 | 1.73E-06 | 1.73E-06 |
| F11 | 4.78E-01 | 4.29E-06 | 1.73E-06 | 2.07E-02 | 1.92E-06 | 1.73E-06 | 1.92E-06 | 1.92E-06 | 1.73E-06 | 6.04E-03 |
| F12 | 1.48E-02 | 7.87E-02 | 1.73E-06 | 2.35E-06 | 1.73E-06 | 1.73E-06 | 1.73E-06 | 6.34E-06 | 1.73E-06 | 1.73E-06 |
| F13 | 2.88E-06 | 3.33E-02 | 1.73E-06 | 1.83E-03 | 1.73E-06 | 1.73E-06 | 1.73E-06 | 9.26E-01 | 1.73E-06 | 2.61E-04 |
| F14 | 4.20E-04 | 3.88E-04 | 1.73E-06 | 2.37E-05 | 1.73E-06 | 1.73E-06 | 8.22E-02 | 3.19E-01 | 1.73E-06 | 4.73E-06 |
| F15 | 7.04E-01 | 1.40E-02 | 1.73E-06 | 1.97E-05 | 1.73E-06 | 1.73E-06 | 3.18E-06 | 2.11E-03 | 1.73E-06 | 3.52E-06 |
| F16 | 2.54E-01 | 1.06E-04 | 1.92E-06 | 7.71E-04 | 1.13E-05 | 1.73E-06 | 5.75E-06 | 1.40E-02 | 1.73E-06 | 1.47E-01 |
| F17 | 1.25E-02 | 6.89E-05 | 1.92E-06 | 4.53E-01 | 7.16E-04 | 1.73E-06 | 1.73E-06 | 3.39E-01 | 1.92E-06 | 3.61E-03 |
| F18 | 1.73E-06 | 3.88E-04 | 1.73E-06 | 1.36E-01 | 1.97E-03 | 1.73E-06 | 9.32E-06 | 1.73E-06 | 1.48E-02 | 2.35E-06 |
| F19 | 2.84E-05 | 1.11E-03 | 1.73E-06 | 2.60E-06 | 1.73E-06 | 1.73E-06 | 2.85E-02 | 3.52E-06 | 1.73E-06 | 4.29E-06 |
| F20 | 3.32E-04 | 2.70E-02 | 1.73E-06 | 1.04E-03 | 1.15E-04 | 1.73E-06 | 4.45E-05 | 4.11E-03 | 1.73E-06 | 5.44E-01 |
| F21 | 2.35E-06 | 2.37E-05 | 1.73E-06 | 6.56E-02 | 1.73E-06 | 1.73E-06 | 1.73E-06 | 5.75E-06 | 1.92E-06 | 3.18E-06 |
| F22 | 1.73E-06 | 6.32E-05 | 1.73E-06 | 2.60E-06 | 4.73E-06 | 1.73E-06 | 1.73E-06 | 1.73E-06 | 1.73E-06 | 1.73E-06 |
| F23 | 1.73E-06 | 1.73E-06 | 1.73E-06 | 1.73E-06 | 1.73E-06 | 1.73E-06 | 2.50E-01 | 1.73E-06 | 1.73E-06 | 1.73E-06 |
| F24 | 1.73E-06 | 2.53E-06 | 1.73E-06 | 1.73E-06 | 1.73E-06 | 1.73E-06 | 1.00E+00 | 2.55E-06 | 1.73E-06 | 1.73E-06 |
| F25 | 1.73E-06 | 1.73E-06 | 1.73E-06 | 1.73E-06 | 3.79E-06 | 1.73E-06 | 1.00E+00 | 1.73E-06 | 1.73E-06 | 1.73E-06 |
| F26 | 1.73E-06 | 1.71E-06 | 1.73E-06 | 1.73E-06 | 1.73E-06 | 1.73E-06 | 1.00E+00 | 1.72E-06 | 1.73E-06 | 1.73E-06 |
| F27 | 1.73E-06 | 1.73E-06 | 1.73E-06 | 1.73E-06 | 1.73E-06 | 1.73E-06 | 2.50E-01 | 1.73E-06 | 1.73E-06 | 1.73E-06 |
| F28 | 1.73E-06 | 1.73E-06 | 1.73E-06 | 1.73E-06 | 1.73E-06 | 1.73E-06 | 2.50E-01 | 1.70E-06 | 1.73E-06 | 1.73E-06 |
| F29 | 1.73E-06 | 1.73E-06 | 1.73E-06 | 1.73E-06 | 1.73E-06 | 1.73E-06 | 5.00E-01 | 1.73E-06 | 1.73E-06 | 1.73E-06 |
| F30 | 1.73E-06 | 1.73E-06 | 1.73E-06 | 1.73E-06 | 1.73E-06 | 1.73E-06 | 1.73E-06 | 1.73E-06 | 1.73E-06 | 1.73E-06 |

**Appendix A.8**．Comparison between BSMA_FBI and alternative GS optimizers on average number of the selected features

| Datasets | Metrics | bGWO | BBA | BGSA | BPSO | bALO | BSSA | bHHO | BFBI | BSMA_FBI |
| --- | --- | --- | --- | --- | --- | --- | --- | --- | --- | --- |
| **Parkinson** | std | **0.52705** | 2.2828 | 0.73786 | 1.1738 | 1.3984 | 1.1595 | 1.792 | 1.5951 | 0.8756 |
|  | avg | **2.5** | 9.1 | 2.9 | 3.4 | 3.2 | 6.7 | 4.1 | 10.1 | 2.9 |
| **penglungEW** | std | 4.1433 | 10.6124 | 8.3193 | 5.7436 | 6.603 | 63.0239 | 13.9666 | 5.5986 | **5.5787** |
|  | avg | 9.5 | 132.8 | 76.9 | 117.9 | 108.4 | 95.3 | 50.8 | 145.7 | **5.7** |
| **clean2** | std | 4.6714 | 7.2541 | 3.5839 | 5.7581 | 6.6742 | 28.6597 | 17.555 | 4.397 | **10.9651** |
|  | avg | 26.6 | 69.2 | 60.8 | 72.4 | 71.1 | 55.4 | 51.8 | 79 | **21.7** |
| **Colon** | std | 25.4296 | 52.764 | 33.0019 | 19.2469 | 23.2866 | 387.5653 | 40.618 | 10.133 | **0.52705** |
|  | avg | 167 | 800.4 | 779.7 | 881 | 857.4 | 716.3 | 235.4 | 960.3 | **1.5** |
| **Leukemia** | std | 47.482 | 179.6424 | 30.0444 | 53.225 | 32.4498 | 1290.3434 | 219.6765 | 35.952 | **4.2635** |
|  | avg | 757.1 | 2866.4 | 3118 | 3339.3 | 3283.9 | 2521 | 831 | 3494.9 | **3.2** |
| **Brain_Tumor1** | std | 37.8983 | 125.6346 | 68.5254 | 56.7963 | 39.5515 | 1333.9267 | 838.6791 | 50.0112 | **5.0596** |
|  | avg | 628.5 | 2538.6 | 2584.2 | 2781.6 | 2724.1 | 1477.5 | 1071.7 | 2910.7 | **4.6** |
| **Brain_Tumor2** | std | 41.9788 | 150.6875 | 92.6864 | 59.1447 | 59.2528 | 1676.3228 | 379.5053 | 41.0649 | **0.70711** |
|  | avg | 1174 | 4231.5 | 4684.1 | 4921.1 | 4860 | 4318 | 1573.5 | 5104.9 | **1.5** |
| **CNS** | std | 41.8968 | 76.0559 | 54.3864 | 54.8717 | 53.0179 | 1036.3078 | 604.4489 | 54.2898 | **1.2293** |
|  | avg | 843.3 | 2981.5 | 3224.9 | 3406.7 | 3330.7 | 2557.9 | 1603.4 | 3525.4 | **1.8** |
| **DLBCL** | std | 22.4044 | 116.4809 | 40.3624 | 26.5255 | 17.6197 | 1040.4524 | 385.4327 | 41.2505 | **2.5408** |
|  | avg | 565.8 | 2293.3 | 2351.3 | 2530.6 | 2495.7 | 1764.2 | 848.8 | 2681.4 | **2.3** |
| **Leukemia1** | std | 38.0526 | 71.4765 | 41.4413 | 37.8424 | 30.4173 | 793.9862 | 191.7468 | 26.3483 | **11.4431** |
|  | avg | 555 | 2157 | 2285.6 | 2467.6 | 2411.9 | 2270.5 | 717.2 | 2609.7 | **11.5** |
| **Leukemia2** | std | 54.6549 | 220.7977 | 57.0536 | 16.0572 | 39.3741 | 2412.007 | 1385.9142 | 20.5969 | **5.5227** |
|  | avg | 1241.4 | 4629.5 | 5038 | 5324.5 | 5264.1 | 3641.4 | 1702.6 | 5514.3 | **7.5** |
| **Lung_Cancer** | std | 63.0312 | 231.6986 | 88.6451 | 69.172 | 51.4376 | 2931.1603 | 1446.2926 | 43.5385 | **44.6816** |
|  | avg | 1507.4 | 5268.3 | 5784.2 | 6048.1 | 5966.4 | 3399.4 | 2350.3 | 6229.6 | **62** |
| **Lungcancer_3class** | std | **0.70711** | 3.6576 | 6.2191 | 2.9833 | 3.5418 | 5.8157 | 5.8538 | 2.6162 | 1.5492 |
|  | avg | **2.5** | 21.4 | 7.3 | 13.7 | 10.1 | 19.6 | 11.4 | 26.8 | 3.2 |
| **Prostate_Tumor** | std | 82.0647 | 512.3306 | 75.9811 | 60.2042 | 55.9175 | 2430.6442 | 588.4224 | 53.7653 | **3.5292** |
|  | avg | 1275.2 | 4047.7 | 4800.7 | 5049.1 | 4959.1 | 3067.1 | 1786.6 | 5198.6 | **5.7** |
| **SRBCT** | std | 16.9036 | 45.8325 | 16.2375 | 30.0372 | 13.6724 | 525.2828 | 126.5498 | 23.2465 | **13.0899** |
|  | avg | 190.2 | 946.2 | 902.1 | 1029.7 | 1000.6 | 605 | 318.2 | 1131.2 | **14.3** |
| **Tumors_9** | std | 57.0584 | 177.3048 | 105.174 | 78.4093 | 40.5906 | 905.8217 | 746.2221 | 39.4456 | **524.0735** |
|  | avg | 682.1 | 2338.1 | 2571.7 | 2729.7 | 2644.4 | 2396.3 | 1599.4 | 2838.2 | **250.1** |
| **Tumors_11** | std | 73.735 | 304.2543 | 97.8984 | 54.7078 | 51.7644 | 2289.2664 | 1359.9563 | 64.0875 | **241.0644** |
|  | avg | 1638.8 | 5157 | 5820.1 | 6091.5 | 5966 | 4371.9 | 3126.3 | 6239.1 | **346.6** |
| **Tumors_14** | std | 132.9782 | 418.4519 | 74.2428 | 65.4897 | 69.0912 | 1979.2153 | 2095.8514 | 68.6279 | **1380.334** |
|  | avg | 2295.1 | 6253.3 | 7293 | 7345.7 | 7324.4 | 6388.1 | 6737.4 | 7502.7 | **1021.4** |
| ARV | | 2.2583 | 5.125 | 4.9639 | 6.9 | 6.1222 | 5.9556 | 3.6167 | 8.7667 | **1.2917** |
| Rank | | 2 | 5 | 4 | 8 | 7 | 6 | 3 | 9 | **1** |

**Appendix A.9**．Comparison between BSMA_FBI and alternative GS optimizers on average error rate

| Datasets | Metrics | bGWO | BBA | BGSA | BPSO | bALO | BSSA | bHHO | BFBI | BSMA_FBI |
| --- | --- | --- | --- | --- | --- | --- | --- | --- | --- | --- |
| **Parkinson** | std | 2.63E-02 | 1.17E-01 | 4.23E-02 | 3.37E-02 | **2.11E-02** | 3.41E-02 | 4.22E-02 | 3.96E-02 | 5.33E-02 |
|  | avg | 3.05E-02 | 1.79E-01 | 3.61E-02 | 1.50E-02 | **1.00E-02** | 1.53E-02 | 3.58E-02 | 4.05E-02 | 5.11E-02 |
| **penglungEW** | std | 0.00E+00 | 1.24E-01 | 5.66E-02 | 3.95E-02 | 4.68E-02 | 6.55E-02 | 0.00E+00 | 6.71E-02 | **0.00E+00** |
|  | avg | 0.00E+00 | 1.24E-01 | 2.68E-02 | 1.25E-02 | 2.22E-02 | 3.10E-02 | 0.00E+00 | 7.46E-02 | **0.00E+00** |
| **clean2** | std | **3.66E-03** | 8.12E-03 | 4.94E-03 | 5.18E-03 | 7.18E-03 | 9.09E-03 | 7.12E-03 | 5.59E-03 | 9.69E-03 |
|  | avg | **3.64E-03** | 4.59E-02 | 9.40E-03 | 2.20E-02 | 1.39E-02 | 3.39E-02 | 2.82E-02 | 2.99E-02 | 3.08E-02 |
| **Colon** | std | 8.61E-02 | 1.39E-01 | 1.52E-01 | 1.04E-01 | 1.04E-01 | 1.36E-01 | 8.47E-02 | 1.91E-01 | **5.27E-02** |
|  | avg | 6.67E-02 | 3.21E-01 | 1.74E-01 | 1.62E-01 | 1.60E-01 | 1.64E-01 | 1.43E-01 | 2.12E-01 | **1.67E-02** |
| **Leukemia** | std | 0.00E+00 | 7.00E-02 | 0.00E+00 | 0.00E+00 | 0.00E+00 | 0.00E+00 | 0.00E+00 | 5.66E-02 | **0.00E+00** |
|  | avg | 0.00E+00 | 6.61E-02 | 0.00E+00 | 0.00E+00 | 0.00E+00 | 0.00E+00 | 0.00E+00 | 2.68E-02 | **0.00E+00** |
| **Brain_Tumor1** | std | 4.68E-02 | 1.22E-01 | 5.46E-02 | 5.75E-02 | 5.75E-02 | 7.15E-02 | 5.46E-02 | 7.08E-02 | **3.51E-02** |
|  | avg | 2.22E-02 | 1.23E-01 | 4.22E-02 | 5.44E-02 | 5.44E-02 | 6.22E-02 | 4.22E-02 | 7.58E-02 | **1.11E-02** |
| **Brain_Tumor2** | std | 9.56E-02 | 3.28E-01 | 1.75E-01 | 1.09E-01 | 1.04E-01 | 1.38E-01 | 1.01E-01 | 1.41E-01 | **0.00E+00** |
|  | avg | 4.50E-02 | 2.43E-01 | 1.00E-01 | 1.02E-01 | 7.83E-02 | 9.43E-02 | 6.17E-02 | 1.77E-01 | **0.00E+00** |
| **CNS** | std | 0.00E+00 | 2.26E-01 | 7.69E-02 | 1.31E-01 | 1.27E-01 | 1.03E-01 | 1.09E-01 | 1.72E-01 | **0.00E+00** |
|  | avg | 0.00E+00 | 2.90E-01 | 4.76E-02 | 1.15E-01 | 1.21E-01 | 1.12E-01 | 9.86E-02 | 2.03E-01 | **0.00E+00** |
| **DLBCL** | std | 0.00E+00 | 1.65E-01 | 3.95E-02 | 3.95E-02 | 3.95E-02 | 5.66E-02 | 3.95E-02 | 3.95E-02 | **0.00E+00** |
|  | avg | 0.00E+00 | 1.00E-01 | 1.25E-02 | 1.25E-02 | 1.25E-02 | 2.68E-02 | 1.25E-02 | 1.25E-02 | **0.00E+00** |
| **Leukemia1** | std | 0.00E+00 | 1.16E-01 | 0.00E+00 | 0.00E+00 | 0.00E+00 | 0.00E+00 | 0.00E+00 | 3.95E-02 | **0.00E+00** |
|  | avg | 0.00E+00 | 1.21E-01 | 0.00E+00 | 0.00E+00 | 0.00E+00 | 0.00E+00 | 0.00E+00 | 1.25E-02 | **0.00E+00** |
| **Leukemia2** | std | 0.00E+00 | 1.37E-01 | 5.66E-02 | 5.66E-02 | 4.52E-02 | 4.52E-02 | 0.00E+00 | 6.23E-02 | **0.00E+00** |
|  | avg | 0.00E+00 | 8.21E-02 | 2.68E-02 | 2.68E-02 | 1.43E-02 | 1.43E-02 | 0.00E+00 | 2.92E-02 | **0.00E+00** |
| **Lung_Cancer** | std | 3.28E-02 | 2.40E-02 | 2.56E-02 | 2.62E-02 | 2.34E-02 | 2.62E-02 | 3.52E-02 | 3.78E-02 | **2.06E-02** |
|  | avg | 1.48E-02 | 6.42E-02 | 1.98E-02 | 2.48E-02 | 1.45E-02 | 2.03E-02 | 2.48E-02 | 4.51E-02 | **9.76E-03** |
| **Lungcancer_3class** | std | 0.00E+00 | 3.81E-01 | 0.00E+00 | 0.00E+00 | 0.00E+00 | 0.00E+00 | 0.00E+00 | 0.00E+00 | **0.00E+00** |
|  | avg | 0.00E+00 | 3.92E-01 | 0.00E+00 | 0.00E+00 | 0.00E+00 | 0.00E+00 | 0.00E+00 | 0.00E+00 | **0.00E+00** |
| **Prostate_Tumor** | std | 3.16E-02 | 1.15E-01 | 4.83E-02 | 5.09E-02 | 6.65E-02 | 6.58E-02 | 6.45E-02 | 1.05E-01 | **3.16E-02** |
|  | avg | 1.00E-02 | 2.16E-01 | 3.00E-02 | 4.82E-02 | 4.82E-02 | 4.73E-02 | 3.73E-02 | 1.28E-01 | **1.00E-02** |
| **SRBCT** | std | 0.00E+00 | 8.59E-02 | 0.00E+00 | 0.00E+00 | 0.00E+00 | 3.95E-02 | 0.00E+00 | 0.00E+00 | **0.00E+00** |
|  | avg | 0.00E+00 | 8.75E-02 | 0.00E+00 | 0.00E+00 | 0.00E+00 | 1.25E-02 | 0.00E+00 | 0.00E+00 | **0.00E+00** |
| **Tumors_9** | std | **0.00E+00** | 2.61E-01 | 7.03E-02 | 5.66E-02 | 6.55E-02 | 6.90E-02 | 1.25E-01 | 1.54E-01 | 3.95E-02 |
|  | avg | **0.00E+00** | 4.07E-01 | 5.40E-02 | 2.68E-02 | 3.10E-02 | 4.21E-02 | 5.83E-02 | 1.60E-01 | 1.25E-02 |
| **Tumors_11** | std | **3.33E-02** | 7.17E-02 | 5.00E-02 | 4.67E-02 | 6.35E-02 | 4.63E-02 | 4.99E-02 | 6.52E-02 | 5.04E-02 |
|  | avg | **1.05E-02** | 1.38E-01 | 3.41E-02 | 3.90E-02 | 5.40E-02 | 5.38E-02 | 4.48E-02 | 8.06E-02 | 5.16E-02 |
| **Tumors_14** | std | **4.88E-02** | 1.17E-01 | 6.79E-02 | 7.20E-02 | 7.48E-02 | 5.87E-02 | 8.01E-02 | 6.08E-02 | 3.91E-02 |
|  | avg | **1.51E-01** | 3.66E-01 | 2.16E-01 | 2.67E-01 | 2.45E-01 | 3.06E-01 | 2.81E-01 | 3.48E-01 | 2.99E-01 |
| ARV | | **3.7806** | 7.3528 | 4.4722 | 4.7778 | 4.6222 | 5.0194 | 4.7611 | 5.875 | 4.3389 |
| Rank | | **1** | 9 | 3 | 6 | 4 | 7 | 5 | 8 | 2 |

**Appendix A.10.** Comparison between BSMA_FBI and alternative GS optimizers on average fitness

| Datasets | Metrics | bGWO | BBA | BGSA | BPSO | bALO | BSSA | bHHO | BFBI | BSMA_FBI |
| --- | --- | --- | --- | --- | --- | --- | --- | --- | --- | --- |
| **Parkinson** | std | 2.50E-02 | 4.65E-02 | 3.99E-02 | 3.13E-02 | **2.12E-02** | 3.13E-02 | 3.95E-02 | 3.75E-02 | 5.00E-02 |
|  | avg | 3.47E-02 | 6.60E-02 | 4.08E-02 | 2.20E-02 | **1.68E-02** | 2.97E-02 | 4.33E-02 | 6.15E-02 | 5.51E-02 |
| **penglungEW** | std | 6.37E-04 | 9.64E-02 | 5.41E-02 | 3.71E-02 | 4.44E-02 | 6.08E-02 | 2.15E-03 | 6.35E-02 | **8.58E-04** |
|  | avg | 1.46E-03 | 8.51E-02 | 3.73E-02 | 3.00E-02 | 3.78E-02 | 4.41E-02 | 7.82E-03 | 9.33E-02 | **8.77E-04** |
| **clean2** | std | **2.84E-03** | 4.49E-03 | 5.33E-03 | 5.50E-03 | 7.23E-03 | 3.64E-03 | 4.79E-03 | 5.05E-03 | 6.37E-03 |
|  | avg | **1.15E-02** | 5.39E-02 | 2.72E-02 | 4.27E-02 | 3.47E-02 | 4.89E-02 | 4.24E-02 | 5.22E-02 | 3.58E-02 |
| **Colon** | std | 8.19E-02 | 1.33E-01 | 1.44E-01 | 9.87E-02 | 9.89E-02 | 1.32E-01 | 7.97E-02 | 1.81E-01 | **5.01E-02** |
|  | avg | 6.75E-02 | 2.33E-01 | 1.85E-01 | 1.76E-01 | 1.73E-01 | 1.74E-01 | 1.42E-01 | 2.25E-01 | **1.59E-02** |
| **Leukemia** | std | 3.33E-04 | 2.86E-03 | 2.11E-04 | 3.73E-04 | 2.28E-04 | 9.05E-03 | 1.54E-03 | 5.38E-02 | **2.99E-05** |
|  | avg | 5.31E-03 | 1.78E-02 | 2.19E-02 | 2.34E-02 | 2.30E-02 | 1.77E-02 | 5.83E-03 | 5.00E-02 | **2.24E-05** |
| **Brain_Tumor1** | std | 4.46E-02 | 6.99E-02 | 5.19E-02 | 5.46E-02 | 5.44E-02 | 7.21E-02 | 5.51E-02 | 6.73E-02 | **3.34E-02** |
|  | avg | 2.64E-02 | 7.06E-02 | 6.19E-02 | 7.52E-02 | 7.47E-02 | 7.16E-02 | 4.92E-02 | 9.66E-02 | **1.06E-02** |
| **Brain_Tumor2** | std | 9.09E-02 | 1.67E-01 | 1.66E-01 | 1.04E-01 | 9.85E-02 | 1.34E-01 | 9.65E-02 | 1.34E-01 | **3.41E-06** |
|  | avg | 4.84E-02 | 1.51E-01 | 1.18E-01 | 1.20E-01 | 9.79E-02 | 1.10E-01 | 6.62E-02 | 1.92E-01 | **7.23E-06** |
| **CNS** | std | 2.94E-04 | 1.16E-01 | 7.30E-02 | 1.25E-01 | 1.21E-01 | 9.57E-02 | 1.04E-01 | 1.63E-01 | **8.62E-06** |
|  | avg | 5.91E-03 | 1.86E-01 | 6.79E-02 | 1.33E-01 | 1.38E-01 | 1.24E-01 | 1.05E-01 | 2.18E-01 | **1.26E-05** |
| **DLBCL** | std | 2.05E-04 | 7.42E-02 | 3.76E-02 | 3.77E-02 | 3.75E-02 | 5.19E-02 | 3.75E-02 | 3.75E-02 | **2.32E-05** |
|  | avg | 5.17E-03 | 4.24E-02 | 3.34E-02 | 3.50E-02 | 3.47E-02 | 4.16E-02 | 1.96E-02 | 3.64E-02 | **2.10E-05** |
| **Leukemia1** | std | 3.57E-04 | 2.17E-03 | 3.89E-04 | 3.55E-04 | 2.86E-04 | 7.45E-03 | 1.80E-03 | 3.74E-02 | **1.07E-04** |
|  | avg | 5.21E-03 | 1.75E-02 | 2.15E-02 | 2.32E-02 | 2.26E-02 | 2.13E-02 | 6.73E-03 | 3.64E-02 | **1.08E-04** |
| **Leukemia2** | std | 2.43E-04 | 8.99E-02 | 5.39E-02 | 5.38E-02 | 4.30E-02 | 3.91E-02 | 6.17E-03 | 5.91E-02 | **2.46E-05** |
|  | avg | 5.53E-03 | 5.68E-02 | 4.79E-02 | 4.92E-02 | 3.70E-02 | 2.98E-02 | 7.58E-03 | 5.23E-02 | **3.34E-05** |
| **Lung_Cancer** | std | 3.10E-02 | 3.83E-02 | 2.43E-02 | 2.50E-02 | 2.23E-02 | 2.78E-02 | 3.18E-02 | 3.59E-02 | **1.96E-02** |
|  | avg | 2.00E-02 | 5.41E-02 | 4.18E-02 | 4.75E-02 | 3.75E-02 | 3.27E-02 | 3.29E-02 | 6.76E-02 | **9.52E-03** |
| **Lungcancer_3class** | std | 6.31E-04 | 2.14E-01 | 5.55E-03 | 2.66E-03 | 3.16E-03 | 5.19E-03 | 5.23E-03 | 2.34E-03 | **1.38E-03** |
|  | avg | 2.23E-03 | 1.37E-01 | 6.52E-03 | 1.22E-02 | 9.02E-03 | 1.75E-02 | 1.02E-02 | 2.39E-02 | **2.86E-03** |
| **Prostate_Tumor** | std | 3.01E-02 | 4.49E-02 | 4.59E-02 | 4.84E-02 | 6.31E-02 | 6.71E-02 | 6.07E-02 | 9.94E-02 | **3.00E-02** |
|  | avg | 1.56E-02 | 1.11E-01 | 5.13E-02 | 6.98E-02 | 6.94E-02 | 5.95E-02 | 4.39E-02 | 1.47E-01 | **9.53E-03** |
| **SRBCT** | std | 3.66E-04 | 4.90E-02 | 3.52E-04 | 6.51E-04 | 2.96E-04 | 4.26E-02 | 2.74E-03 | 5.04E-04 | **2.84E-04** |
|  | avg | 4.12E-03 | 3.47E-02 | 1.95E-02 | 2.23E-02 | 2.17E-02 | 2.50E-02 | 6.89E-03 | 2.45E-02 | **3.10E-04** |
| **Tumors_9** | std | **4.98E-04** | 1.67E-01 | 6.72E-02 | 5.40E-02 | 6.21E-02 | 6.85E-02 | 1.19E-01 | 1.47E-01 | 3.85E-02 |
|  | avg | **1.41E-02** | 1.41E-02 | 1.41E-02 | 1.41E-02 | 1.41E-02 | 1.41E-02 | 1.41E-02 | 1.41E-02 | 1.41E-02 |
| **Tumors_11** | std | **3.16E-02** | 7.29E-02 | 4.76E-02 | 4.44E-02 | 6.02E-02 | 4.03E-02 | 4.73E-02 | 6.20E-02 | 4.74E-02 |
|  | avg | **1.65E-02** | 1.06E-01 | 5.56E-02 | 6.13E-02 | 7.51E-02 | 6.85E-02 | 5.51E-02 | 1.01E-01 | 5.04E-02 |
| **Tumors_14** | std | **4.61E-02** | 7.67E-02 | 6.43E-02 | 6.82E-02 | 7.11E-02 | 5.65E-02 | 7.47E-02 | 5.77E-02 | 3.82E-02 |
|  | avg | **1.51E-01** | 3.37E-01 | 2.30E-01 | 2.78E-01 | 2.58E-01 | 3.12E-01 | 2.89E-01 | 3.55E-01 | 2.87E-01 |
| ARV | | 2.4167 | 6.1639 | 4.7556 | 6.1583 | 5.5667 | 5.8917 | 4.175 | 7.8528 | **2.0194** |
| Rank | | 2 | 8 | 4 | 7 | 5 | 6 | 3 | 9 | **1** |

**Appendix A.11.** Comparison between BSMA_FBI and alternative GS optimizers on average computational time

| Datasets | Metrics | bGWO | BBA | BGSA | BPSO | bALO | BSSA | bHHO | BFBI | BSMA_FBI |
| --- | --- | --- | --- | --- | --- | --- | --- | --- | --- | --- |
| **Parkinson** | std | 0.57742 | 0.04671 | 0.0554 | 0.048538 | **0.02579** | 0.050169 | 0.073774 | 0.30768 | 0.10639 |
|  | avg | 7.2428 | 1.9453 | 2.0105 | 1.8994 | **1.8948** | 1.9779 | 3.2121 | 6.9865 | 5.6239 |
| **penglungEW** | std | 0.068776 | 0.084986 | 0.092661 | 0.064572 | **0.095399** | 0.072618 | 0.099266 | 0.74226 | 0.20154 |
|  | avg | 3.2053 | 2.3683 | 2.6521 | 2.2763 | **2.1766** | 2.1937 | 3.7546 | 7.915 | 7.4196 |
| **clean2** | std | **14.6589** | 19.6179 | 11.021 | 20.4229 | 12.5681 | 9.2869 | 35.7911 | 53.0437 | 331.2897 |
|  | avg | **179.5979** | 410.6196 | 371.0988 | 404.5282 | 403.179 | 422.0486 | 826.2436 | 2818.8039 | 2026.1654 |
| **Colon** | std | 0.088306 | 0.17737 | 0.21112 | 0.078231 | **0.11456** | 0.065728 | 0.19232 | 0.90805 | 0.67318 |
|  | avg | 9.4886 | 3.4395 | 5.902 | 3.2169 | **3.0448** | 3.2147 | 5.5531 | 13.3382 | 12.9233 |
| **Leukemia** | std | 0.28411 | 0.46999 | 0.59261 | 0.25089 | **0.42255** | 0.35484 | 0.61423 | 5.6764 | 2.0064 |
|  | avg | 29.8522 | 10.0909 | 20.3978 | 10.0547 | **10.0373** | 10.3323 | 17.4185 | 52.1253 | 49.4081 |
| **Brain_Tumor1** | std | 0.25881 | 0.33952 | 0.60418 | 0.41543 | **0.21087** | 0.46486 | 0.89375 | 6.7469 | 6.4385 |
|  | avg | 25.9164 | 11.0949 | 18.9952 | 10.7673 | **10.5865** | 11.0066 | 19.2135 | 57.3053 | 44.9394 |
| **Brain_Tumor2** | std | 0.32193 | 0.44308 | 0.73845 | 0.37007 | **0.40558** | 0.40004 | 1.0761 | 3.7263 | 4.7108 |
|  | avg | 42.0069 | 10.6531 | 26.2177 | 10.5977 | **9.8919** | 10.4781 | 17.4813 | 51.4039 | 45.9882 |
| **CNS** | std | 0.17016 | 0.50923 | 0.43946 | 0.41868 | **0.1326** | 0.22101 | 0.62118 | 2.8403 | 4.8162 |
|  | avg | 29.6598 | 9.1375 | 19.2386 | 8.8339 | **8.2992** | 8.7524 | 15.2723 | 48.0777 | 33.0326 |
| **DLBCL** | std | 0.14723 | 0.48041 | 0.58123 | 0.29736 | **0.40554** | 0.31494 | 0.51473 | 4.5114 | 2.2363 |
|  | avg | 23.5111 | 9.0102 | 16.0367 | 8.4167 | **8.4091** | 8.9198 | 14.7874 | 45.4331 | 40.2831 |
| **Leukemia1** | std | 0.15518 | 0.22057 | 0.30177 | 0.2888 | **0.14447** | 0.19694 | 0.36135 | 2.9686 | 2.9063 |
|  | avg | 22.8439 | 8.1924 | 15.4122 | 8.1178 | **7.8329** | 8.3243 | 13.6198 | 42.9575 | 37.5125 |
| **Leukemia2** | std | 0.398 | 0.60511 | 1.0263 | 0.53379 | **0.30691** | 0.65944 | 1.0311 | 5.1434 | 8.4902 |
|  | avg | 46.6861 | 15.5224 | 32.5822 | 15.511 | **14.6202** | 15.276 | 25.6764 | 82.0338 | 75.1888 |
| **Lung_Cancer** | std | 1.0453 | 2.0029 | 2.1367 | 1.4317 | **1.4448** | 2.3155 | 7.3864 | 30.528 | 49.1476 |
|  | avg | 69.7209 | 63.7264 | 80.544 | 64.0032 | **63.3865** | 63.9256 | 116.8407 | 402.2769 | 231.6315 |
| **Lungcancer_3class** | std | 0.020581 | 0.025404 | 0.043072 | 0.047564 | **0.037896** | 0.040493 | 0.1149 | 0.25905 | 0.084908 |
|  | avg | 1.9987 | 1.8492 | 1.8921 | 1.8221 | **1.788** | 1.7935 | 3.0606 | 6.5821 | 5.5277 |
| **Prostate_Tumor** | std | 0.45631 | 0.51001 | 0.70413 | 0.6812 | **0.70651** | 0.65539 | 0.75011 | 13.0906 | 13.3363 |
|  | avg | 46.5649 | 20.7926 | 36.4692 | 21.2756 | **20.2109** | 21.1205 | 36.7295 | 126.7188 | 82.5586 |
| **SRBCT** | std | 0.07617 | 0.18709 | 0.14381 | 0.094654 | **0.12114** | 0.15951 | 0.26556 | 1.2898 | 0.75161 |
|  | avg | 11.0394 | 4.3538 | 7.174 | 4.1195 | **3.9966** | 4.219 | 7.1854 | 23.7921 | 22.1003 |
| **Tumors_9** | std | 0.17764 | 0.3001 | 0.45235 | 0.44858 | **0.22883** | 0.39593 | 1.1286 | 3.1164 | 4.5204 |
|  | avg | 24.4772 | 7.6689 | 15.4146 | 7.644 | **7.3116** | 7.7641 | 13.0202 | 39.7427 | 30.5479 |
| **Tumors_11** | std | 0.80683 | **1.6273** | 2.7147 | 1.7315 | 1.1588 | 1.6655 | 6.4992 | 20.826 | 32.511 |
|  | avg | 66.484 | **49.8553** | 68.195 | 51.1068 | 50.4669 | 51.3407 | 95.5546 | 331.9326 | 133.9563 |
| **Tumors_14** | std | **2.5147** | 7.1015 | 5.8749 | 5.61 | 5.1663 | 5.5211 | 19.537 | 49.2386 | 78.253 |
|  | avg | **118.7483** | 144.5427 | 168.9375 | 149.3442 | 148.5027 | 148.0644 | 303.0378 | 1004.1516 | 222.7412 |
| ARV | | 6.1722 | 3.0444 | 5.4778 | 2.7389 | **1.9556** | 2.9833 | 5.8667 | 8.8444 | 7.9167 |
| Rank | | 7 | 4 | 5 | 2 | **1** | 3 | 6 | 9 | 8 |
